# LncRNA Foxo6os as a Novel “ Scaffold” Mediates MYBPC3 in Combating Pathological Cardiac Hypertrophy and Heart Failure

**DOI:** 10.1002/advs.202507365

**Published:** 2025-06-23

**Authors:** Jie Sheng, Qin Lin, Yizhuo Sun, Yilei Meng, Sangyu Hu, Huaming Cao, Fang Lin, Yuping Zhu, Luying Peng, Li Li

**Affiliations:** ^1^ State Key Laboratory of Cardiology and Medical Innovation Center, Shanghai East Hospital, School of Medicine Tongji University Shanghai 200120 China; ^2^ Shanghai Arrhythmias Research Center Shanghai East Hospital Tongji University School of Medicine Shanghai 200120 China; ^3^ Stem Cell Research Center Medical School Tongji University Shanghai 200120 China; ^4^ Department of Cardiology Shibei Hospital Shanghai 200120 China; ^5^ Department of Cell and Genetics Tongji University School of Medicine Shanghai 200120 China

**Keywords:** cardiomyocyte contractile, heart failure, lncRNA Foxo6os, MYBPC3, PKC‐α

## Abstract

Heart failure (HF) as the terminal stage of various cardiac diseases, its underlying molecular mechanisms still remain elusive. Emerging evidence have implicated long noncoding RNAs (lncRNAs) play a multifaceted role in the progression of cardiac hypertrophy and HF. Here, it is identified that a lncRNA forkhead box O6, opposite strand (Foxo6os) is significantly downregulated in murine HF model induced using transverse aortic constriction (TAC). Knockdown of Foxo6os accelerates cardiomyocyte hypertrophy, reflects as elevated expression of atrial natriuretic peptide (ANP), brain natriuretic peptide (BNP), and myosin heavy chain 7 (MYH7). Conversely, Foxo6os overexpression can improve cardiac function and alleviate adverse cardiac remodeling. Mechanistically, Foxo6os directly interacts with myosin‐binding protein‐C (MYBPC3), which then recruits protein kinase C alpha (PKC‐α) to facilitate MYBPC3 phosphorylation, resulting in maintaining myocardial contractility and postponing HF progression. Therefore, these findings underscore the critical role of Foxo6os in preserving cardiomyocyte contractile function, suggesting a potential for Foxo6os as a novel therapeutic target of HF.

## Graphical abstract

Schematic overview showing that forkhead box O6, opposite strand (Foxo6os) acts as a “scaffold”, directly binding myosin‐binding protein‐C (MYBPC3) and recruiting protein kinase C (PKC‐α) to mediate site‐specific phosphorylation of MYBPC3. This post‐translational modification supports cardiac contraction by regulating L‐type Ca^2+^ channels, especially under myocardial hypertrophy‐induced heart failure (HF).

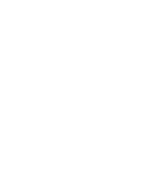

## 1 Introduction

Heart failure (HF) represents the ultimate outcome of diverse cardiovascular diseases, characterized by a diminished cardiac output insufficient to meet the body's metabolic demands.^[^ ^1^ ^]^ Despite advancements in treatment modalities and pharmaceutical approaches have improved patient survival over the decades, prognosis still remains poor,^[^ ^2^ ^]^ which has emerged as a significant challenge across the domains of healthcare, society, and economics.^[^ ^3^, ^4^ ^]^ Therefore, further understanding the intricate regulatory mechanisms underlying HF and pathological myocardial remodeling for finally identifying potential drug targets is crucial for clinical interventions.

The limited capacities in differentiation and proliferation of cardiomyocytes make them difficult to cope with myocardial injury factors, such as prolonged mechanical stress, blood pressure overload, and pathological stimuli, which usually induce irreversible compensatory cardiac hypertrophy, eventually may lead to HF.^[^ ^5^ ^]^ Given the absence of a specific therapeutic intervention, the pathological hypertrophy of the myocardium as a major driving factor influences both the prevalence and lethality of HF.^[^ ^6^ ^]^ Thus, the activation of endogenous protective mechanisms within the myocardium represents a pivotal strategy in the comprehensive management of HF.

Long non‐coding RNAs (LncRNAs) as a class of endogenous regulatory molecules underscore the diverse roles across a range of biological processes and disease mechanisms,^[^ ^7^, ^8^ ^]^ including the modulation of nearby gene transcription via *cis‐* and *trans‐*acting ways.^[^ ^9^, ^10^ ^]^ Recent studies have illuminated the growing interest in the interplay between lncRNAs and proteins,^[^ ^11^, ^12^ ^]^ particularly within the cardiovascular field.^[^ ^13^, ^14^ ^]^ For example, lncRNA CAIF can engage with the p53/myocardin‐dependent autophagy pathway through its binding to p53 during the cardiomyocyte autophagy.^[^ ^15^ ^]^ Overexpression of lncRNA CCRR promotes an interaction with CIP85, which in turn impedes CX43 endocytosis and slows cardiac arrhythmias.^[^ ^16^ ^]^ In dilated cardiomyopathy (DCM), the energy metabolism‐related lncRNA CHKB‐DT interacts with ALDH2 mRNA and FUS via the GGUG motif. Knockdown of CHKB‐DT accelerates ALDH2 mRNA degradation and enhances the 4‐hydroxy‐2‐nonenal (4‐HNE) accumulation, implicating its potential as a therapeutic target for DCM.^[^ ^17^ ^]^ These results suggest crucial regulation of lncRNAs in cardiac development and pathophysiology.

Based on analysis for a multitude of high‐throughput transcriptomic datasets derived from mouse HF samples, we here observed a significant downregulation of a lncRNA, forkhead box O6, opposite strand (Foxo6os) in cardiac hypertrophy models. Previous studies have showed that forkhead box O6 (Foxo6) plays a critical protective role in cardiovascular diseases by modulating cellular metabolism, inflammatory responses, and apoptosis.^[^ ^18^ ^]^ Foxo6os, an antisense lncRNA located upstream of the Foxo6 gene,^[^ ^19^ ^]^ shows high expression in cardiac tissue annotated in AnnoLnc2^[^ ^20^ ^]^ and Expression Atlas,^[^ ^21^ ^]^ suggesting its potential role in the regulation of cardiac function, particularly during cardiac hypertrophy. We really found that Foxo6os transcription is markedly reduced in the cardiac hypertrophy model. However, the molecular details that how Foxo6os contributes to the pathogenesis of HF induced by cardiac hypertrophy are not yet elucidated.

Myosin‐binding protein‐C (MYBPC3), the cardiac isoform, is an integral sarcomere component that exerts a crucial role in modulating cardiac muscle contractility^[^ ^22^, ^23^ ^]^ through engaging its N‐terminal domains with both the actin‐rich thin filaments and the myosin‐laden thick filaments, thereby governing their regulatory states.^[^ ^24^ ^]^ Physiologically, phosphorylation at specific sites within the cardiac‐specific M‐motif of MYBPC3 plays a pivotal role in governing its interaction with both the thin and thick filament systems.^[^ ^25^, ^26^ ^]^ This intricate process is orchestrated by multiple of enzymes, including protein kinase A (PKA), protein kinase C (PKC), protein kinase D (PKD), ribosomal S6 kinase (RSK2), Ca^2+^/calmodulin‐dependent protein kinase II (CaMKII), and glycogen‐synthetase kinase 3 (GSK3).^[^ ^27^, ^28^ ^]^ Notably, MYBPC3 phosphorylation is significantly reduced during HF, indicating a vital role in cardiac dysfunction.^[^ ^29^ ^]^ Consistent with this, ablation of MYBPC3 phosphorylation in animal models results in cardiomyopathy and ultimately to HF.^[^ ^28^ ^]^ Additionally, we detected an interplay between Foxo6os and MYBPC3, with a distinct correlation in their transcriptomic profiles. However, whether Foxo6os directly interacts with MYBPC3 and affects on the pathogenesis of HF is largely unknown.

In this study, we demonstrate that Foxo6os interacts with MYBPC3 to regulate its expression, and mediate PKC‐α for phosphorylation modification, which exerted a maintaining role on the function of MYBPC3. Dysregulation of Foxo6os expression impact the phosphorylation level of MYBPC3, leading to compromised cardiac contractility and ultimately contributing to HF. Therefore, our results elucidate a previously unrecognized role of Foxo6os in the progression of HF.

## 2 Results

### 2.1 LncRNA Foxo6os is Downregulated in Mouse Heart with HF

**Figure 1:**
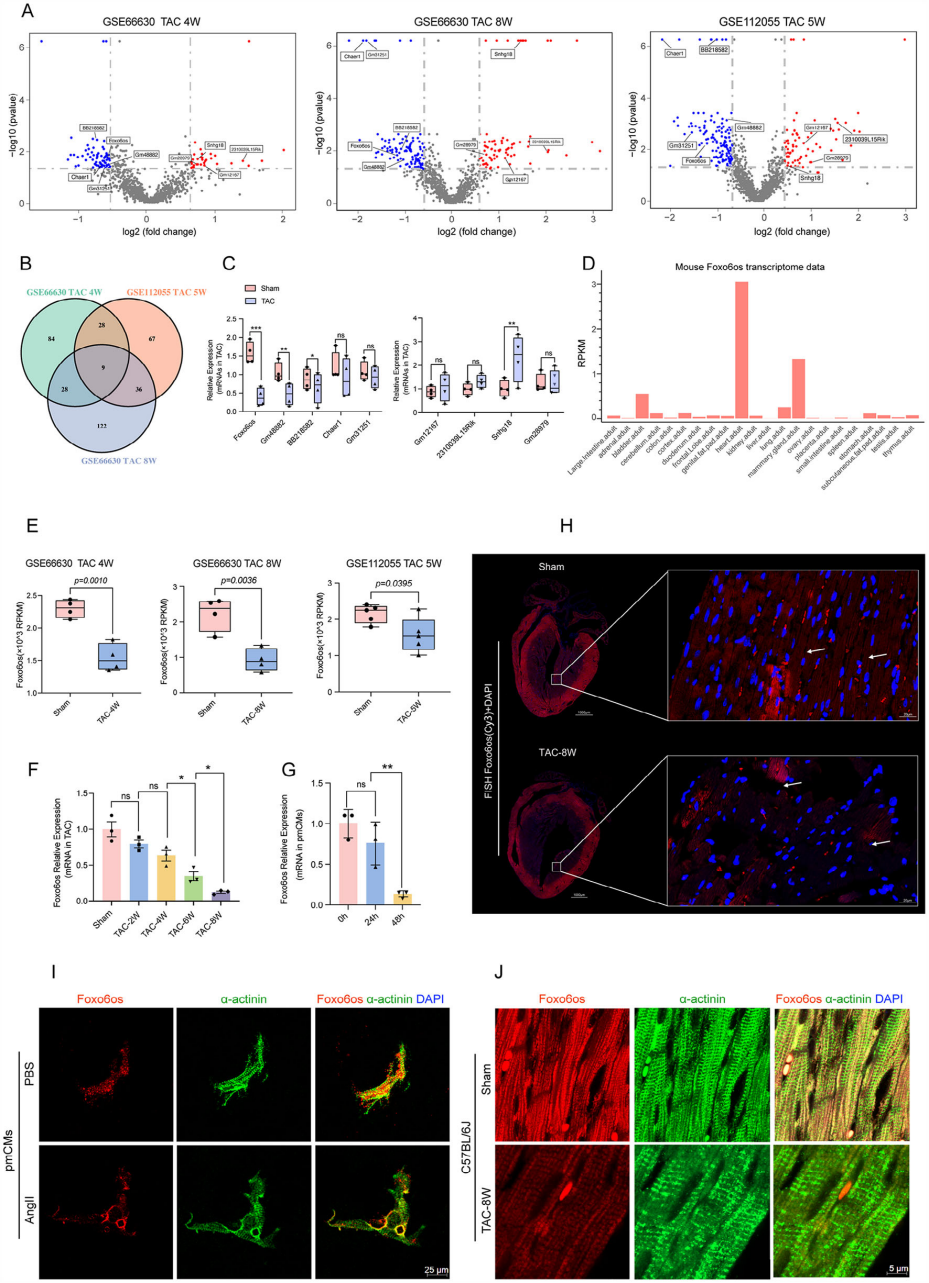
Foxo6os is downregulated in HF. A,B) The differentially expressed genes (DEGs) from the hearts of mice that underwent sham surgery or different stages of TAC‐induced HF (from GSE66630 at 4, 8 W, and GSE112055 at 5 W) were showed by volcano plot(A) and Venn diagram(B). C) RT‐qPCR analysis of DEGs in 8 W after TAC surgery (*n* = 4/group). D) Expression levels of Foxo6os across various tissues in mice. The data was sourced from the Mouse ENCODE transcriptome data (PMID: 25409824). E) The transcriptional level of Foxo6os in GSE66630 and GSE112055 datasets. F) RT‐qPCR analysis of Foxo6os expression demonstrated a gradual decline following TAC surgery, ultimately rendering it undetectable by TAC‐8 W (*n* = 3/group). G) RT‐qPCR assays showed a consistent decline in the expression levels of Foxo6os over time in pmCMs, following the induction of HF through pathological myocardial hypertrophy caused by continuous administration of Ang II(10 nM) for 48 h (*n* = 3/group). H) Representative image of FISH staining in mice heart at 8 W post‐TAC surgery and sham‐operated group (*n* = 5/group, Scale bar = 1000 µm). I) Representative immunofluorescence of Foxo6os/α‐actinin/DAPI in PBS and Ang II (10 nM)‐treated pmCMs (*n* = 3/group, Scale bar = 25 µm). J) Representative immunofluorescence of Foxo6os/α‐actinin/DAPI in heart from sham and TAC mice hearts (*n* = 3/group, Scale bar = 5 µm). All experiments were performed with more than three independent replicates, ns = not significant, *p < 0.05, ** *p* < 0.01, *** *p* < 0.001. These data are presented as means ± SD and analyzed using unpaired Student's *t‐*test.

To explore the potential role of lncRNAs in HF progression, we analyzed RNA‐seq dataset (GSE66630 and GSE112055) and identified 9 overlapping differentially expressed lncRNAs across 3 distinct stages of cardiac hypertrophy (**Figure** **1**A,B). Among these Foxo6os exhibited significant downregulation in the transverse aortic constrictio (TAC)‐induced 8‐week (8 W) HF mouse model compared with the sham group (Figure 1C). Given its cardiac‐specific enrichment^[^ ^30^ ^]^ (Figure 1D), this dysregulation suggests a potential role in pathological cardiac remodeling. Further analysis of TAC samples from GSE66630 and GSE112055 confirmed progressive Foxo6os suppression during HF progression, with expression becoming undetectable by the 8 W timepoint (Figure 1E,F). Consistent with in vivo findings, angiotensin II (Ang II)‐treated neonatal mouse left ventricular cardiomyocytes recapitulated Foxo6os downregulation (Figure 1G), reinforcing its association with hypertrophic stress. Fluorescence in situ hybridization (FISH) analysis revealed that Foxo6os was primarily expressed in the ventricular wall, displaying continuous expression. However, in the TAC‐8 W model, Foxo6os expression was significantly reduced and fragmented (Figure 1H). Further analysis revealed that Foxo6os exhibits co‐localization with the sarcomere structure under physiological conditions, with its expression pattern showing a continuous distribution that aligns with the periodic arrangement of the sarcomere. In both the TAC‐8 W model and Ang II‐induced cardiomyocyte hypertrophy model, as the sarcomere structure undergoes pathological disarray, this characteristic co‐localization expression pattern was lost (Figure 1I,J). Collectively, these results implicate that Foxo6os may serve as a potential regulator of pathological processes of HF, potentially through mechanisms linked to sarcomere integrity and stress‐responsive transcriptional control.

### 2.2 Cardiac‐Specific Overexpression of Foxo6os Attenuates TAC‐Induced HF in Mice

**Figure 2:**
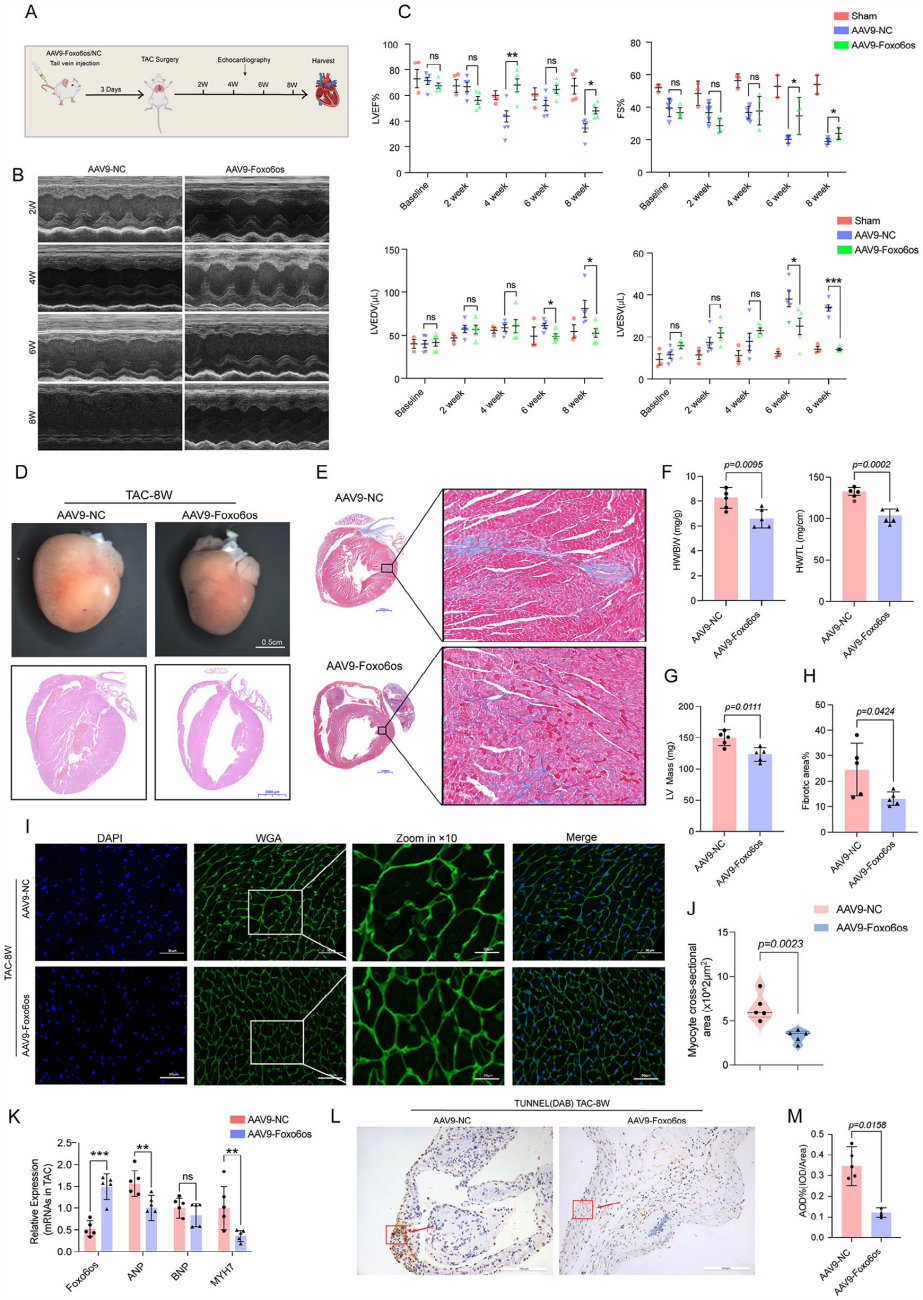
Foxo6os exhibits a notable inhibitory effect on the progression of HF in mice. A) Schematic representation of AAV9‐Foxo6os/NC injection following TAC surgery. B) Representative echocardiographic images of AAV9‐NC and AAV9‐Foxo6os mice at 2, 4, 6, and 8 W post‐TAC surgery. C) The echo parameters of sham(*n* = 3), AAV9‐NC(*n* = 5), and AAV9‐Foxo6os (*n* = 5) mice were calculated at baseline, as well as at 2, 4, 6, and 8 W post‐TAC surgery. D) Representative features of the heart (top, Scale bar = 0.5 cm) and hematoxylin and eosin (H&E)–stained images of cardiac morphology (bottom, Scale bar = 2000 µm) respectively in AAV9‐NC and AAV9‐Foxo6os at 8 W post‐TAC surgery mice (*n* = 5/group). E) Representative Masson staining images, as well as partial magnified view in AAV9‐NC and AAV9‐Foxo6os groups (*n* = 5/group, Scale bar = 1000 µm). F) Comparison of HW/BW and HW/TL between AAV9‐NC and AAV9‐Foxo6os groups at 8 W post‐TAC surgery mice (*n* = 5/group). G) Left ventricular mass (LV Mass) was detected in AAV9‐NC and AAV9‐Foxo6os groups of post‐TAC surgery 8 W mice (*n* = 5/group). H) Quantitation of cardiac fibrosis in AAV9‐NC and AAV9‐Foxo6os groups (*n* = 5/group). I,J) Representative WGA staining images, as well as partial magnified view, and quantitation of cardiomyocyte sizes in AAV9‐NC and AAV9‐Foxo6os groups of post‐TAC surgery 8 W mice (*n* = 5/group, Scale bar = 50 µm). K) RT‐qPCR analysis showing ANP, BNP and MYH7 expression in AAV9‐NC and AAV9‐Foxo6os group at 8 W post‐TAC surgery mice (*n* = 5/group). L,M) Representative TUNEL staining was performed to assess apoptosis, along with quantification of DAB‐positive staining in heart sections from AAV9‐NC and AAV9‐Foxo6os groups of post‐TAC surgery 8 W mice (*n* = 5/group). All experiments were performed with more than three independent replicates. ns = not significant, * *p* < 0.05, ** *p* < 0.01, *** *p* < 0.001. These data are presented as means ± SD and analyzed using unpaired Student's *t*‐test.

To elucidate the functional role of Foxo6os in cardiac hypertrophy, we established TAC‐8 W mice model that showed progressive contractile dysfunction characterized by reduced left ventricular ejection fraction (LVEF) and fraction shortening (FS), along with increased of left ventricular end‐diastolic/systolic diameter (LVEDV/LVESV) (Figure S1A,B, Supporting Information). Consistently, the TAC group exhibited distinctly enlarged heart size compared to the sham group (Figure S1C, Supporting Information), accompanied by elevated left ventricular mass (LV Mass), and increased heart weight‐to‐body weight (HW/BW) and heart weight‐to‐tibia length (HW/TL) ratios (Figure S1D, Supporting Information). The mRNA level upregulation of hypertrophy markers including atrial natriuretic peptide (ANP), B‐type natriuretic peptide (BNP), and myosin heavy chain 7 (MYH7) in the TAC‐8 W mice model (Figure S1E, Supporting Information), which also displayed increased cardiomyocyte cross‐sectional area and enhanced interstitial and perivascular fibrosis (Figure S1F,G,H,I, Supporting Information). Next, we challenged the mouse model by overexpression of Foxo6os mediated with the serotype 9 adeno‐associated virus (AAV9) carrying the cardiac troponin T (cTnT) promoter, followed with TAC surgery (**Figure** **2**A). Echocardiographic assessments indicated that Foxo6os overexpression alleviated TAC‐induced pathological cardiac hypertrophy compared with control mice, as indicated by improved LVEF and FS and reducing LVESV/LVEDV (Figure 2B,C), and also reduced heart size and LV Mass, diminished ventricular wall thickness, and decreased HW/TL and HW/BW ratios (Figure 2D,F,G). Moreover, the forced‐expressing Foxo6os reduced cardiomyocyte cross‐sectional area (Figure 2I,J) and attenuated TAC‐induced cardiac fibrosis (Figure 2E,H), while consistently downregulating hypertrophic markers ANP, BNP, and MYH7 (Figure 2K). Intriguingly, the TUNEL assay showed lower apoptosis rates in the present of Foxo6os overexpression (Figure 2L,M). Following the delivery of Foxo6os/NC via the AAV9 vector driven by the cTnT promoter, the experimental group exhibited targeted overexpression of Foxo6os in cardiac tissue. In contrast, expression levels in non‐targeted organs such as the lung, kidney, and stomach remained low across both the experimental and control groups, with no significant differences observed between them. Importantly, beyond the aforementioned organs, no detectable Foxo6os expression was found in other tissues, including the liver, spleen, or peripheral blood (Figure S1J, Supporting Information). All of the evidence indicate Foxo6os really involved in the progression of TAC‐induced HF.

### 2.3 Cardiac‐Specific Knockdown of Foxo6os Exacerbates TAC‐Induced HF in Mice

**Figure 3:**
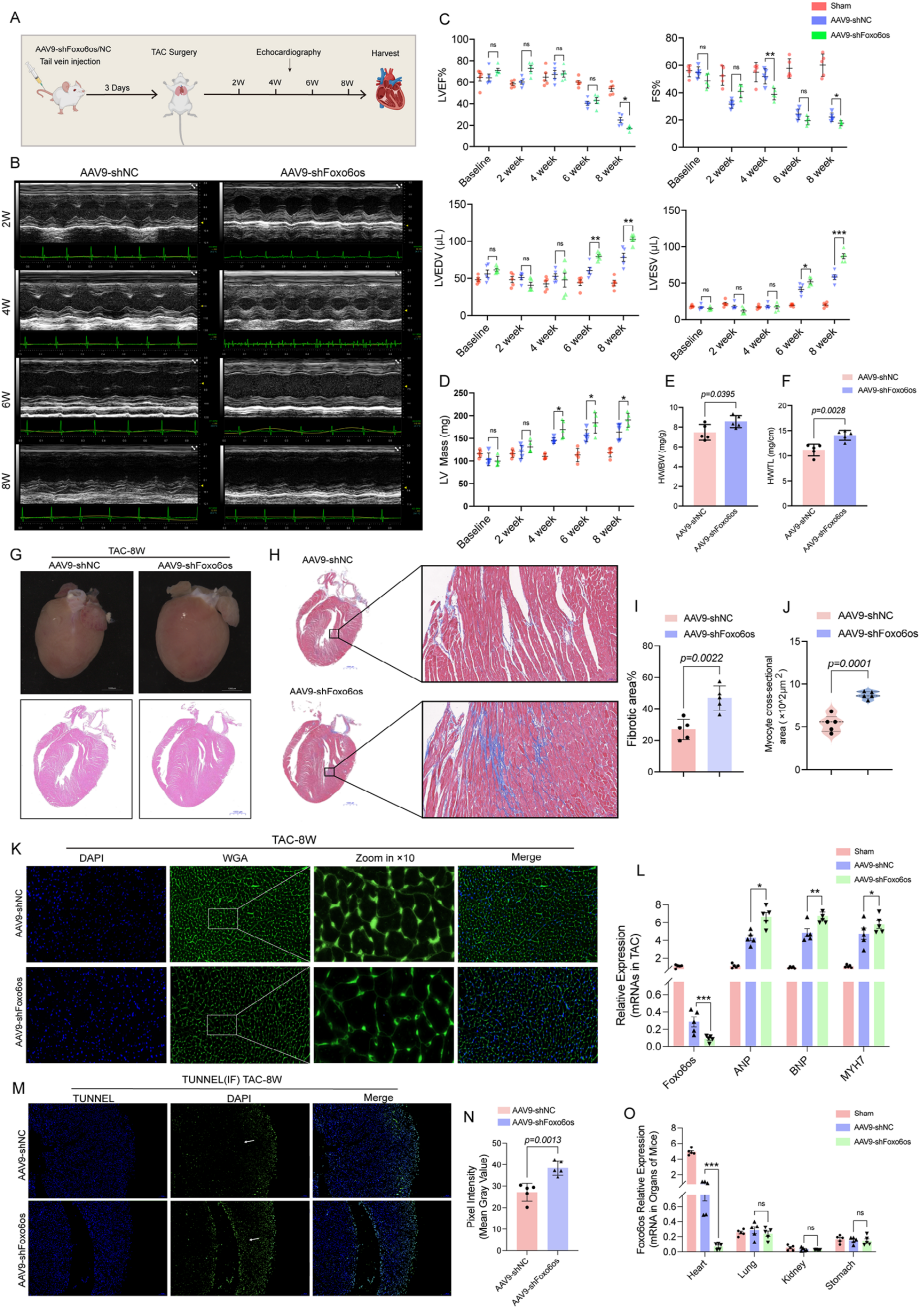
Cardiac‐specific knockdown of Foxo6os accelerates the pathological progression of HF in mice. A) Schematic representation of AAV9‐shNC/Foxo6os injection following TAC surgery. B) Representative echocardiographic images of AAV9‐shNC and AAV9‐shFoxo6os mice at 2, 4, 6, and 8 W post‐TAC surgery. C,D) The echo parameters of sham, AAV9‐shNC, and AAV9‐shFoxo6os mice were calculated at baseline, 2, 4, 6, and 8 W post‐TAC surgery (*n* = 5/group). E,F) Comparison of HW/BW and HW/TL between AAV9‐shNC and AAV9‐shFoxo6os groups of 8 W post‐TAC surgery mice (*n* = 5/group). G) Representative features of the heart (top, Scale bar = 1000 µm), and H&E–stained images of cardiac morphology (bottom, Scale bar = 1000 µm) respectively in AAV9‐shNC and AAV9‐shFoxo6os groups of 8 W post‐TAC surgery mice (*n* = 5/group). H,I) Representative Masson staining images, as well as partial magnified view, and quantitation of cardiac fibrosis in AAV9‐shNC and AAV9‐shFoxo6os groups of 8 W post‐TAC surgery mice (*n* = 5/group, Scale bar = 1000 µm). J,K) Representative WGA staining images, as well as partial magnified view, and quantitation of cardiomyocyte sizes in AAV9‐shNC and AAV9‐shFoxo6os groups of 8 W post‐TAC surgery mice (*n *= 5/group). L) RT‐qPCR analysis showing ANP, BNP and MYH7 expression in AAV9‐shNC and AAV9‐shFoxo6os groups of 8 W post‐TAC surgery mice (*n* = 5/group). M,N) Representative TUNEL‐IF staining was performed to assess apoptosis, along with quantification of TUNNEL‐positive staining in heart sections from AAV9‐shNC and AAV9‐shFoxo6os groups of post‐TAC surgery 8 W mice (*n* = 5/group). O) RT‐qPCR analysis showing the Foxo6os expression in the organs of sham, AAV9‐shNC and AAV9‐shFoxo6os groups (*n* = 5/group). ns = not significant, *p < 0.05, **p < 0.01, ***p < 0.001. These data are presented as means ± SD and analyzed using unpaired Student's *t*‐test.

To further investigate whether cardiac‐specific knockdown of Foxo6os exacerbates TAC‐induced ventricular remodeling and subsequently accelerates the pathological progression of HF, mice were first injected with AAV9 carrying the cardiac‐specific promoter cTnT and either a Foxo6os interference sequence (AAV9‐shFoxo6os) or negative control sequence (AAV9‐shNC) via tail vein injection, followed by TAC surgery 3 days later (**Figure** **3**A). Echocardiographic analysis showed that Foxo6os knockdown aggravated TAC‐induced cardiac pathological hypertrophy, as demonstrated by decreased LVEF and FS, along with the increase in LVESV/LVEDV compared to the controls (Figure 3B,C). Notably, AAV9‐shFoxo6os mice exhibited a more pronounced increase in left ventricular mass from 2 to 8 W post‐TAC compared to the AAV9‐shNC group (Figure 3D). Both HW/BW and HW/TL ratios were significantly elevated in Foxo6os‐knockdown TAC‐8 W mice (Figure 3E,F). Further analysis demonstrated that the AAV9‐shFoxo6os‐specific intervention really resulted in pathological cardiac remodeling in the TAC‐8 W mice. Histological analysis confirmed that Foxo6os suppression led to a significant increase in heart size and a marked thickening of the left ventricular wall by suppressing Foxo6os compared to the control group (Figure 3G). Masson's trichrome staining also shown a significant aggravation in the cardiac fibrosis of the left ventricular myocardium (Figure 3H,I). Additionally, the cross‐sectional area of cardiomyocytes was obvious enlargement (Figure 3J,K). The RT‐qPCR analysis confirmed successful Foxo6os knockdown at the mRNA level and consequent of increased cardiac remodeling markers ANP, BNP, and MYH7 (Figure 3L). Furthermore, the aggravation of apoptosis in cardiomyocytes was also triggered by interfering Foxo6os expression (Figure 3M,N). Through analysis of the organ expression status of Foxo6os, we found that Foxo6os was predominantly expressed in the cardiac tissue, with minimal detection in the lung, kidney, or stomach, and no expression in the liver, spleen, and peripheral blood (Figure 3O). Importantly, AAV9‐mediated Foxo6os knockdown specifically targeted cardiac tissue without affecting Foxo6os levels in other organs. All of these results indicate that Foxo6os is a key regulator of pressure overload‐induced HF.

### 2.4 Foxo6os Overexpression Alleviates AngII‐Induced Cardiac Hypertrophy In Vitro

**Figure 4:**
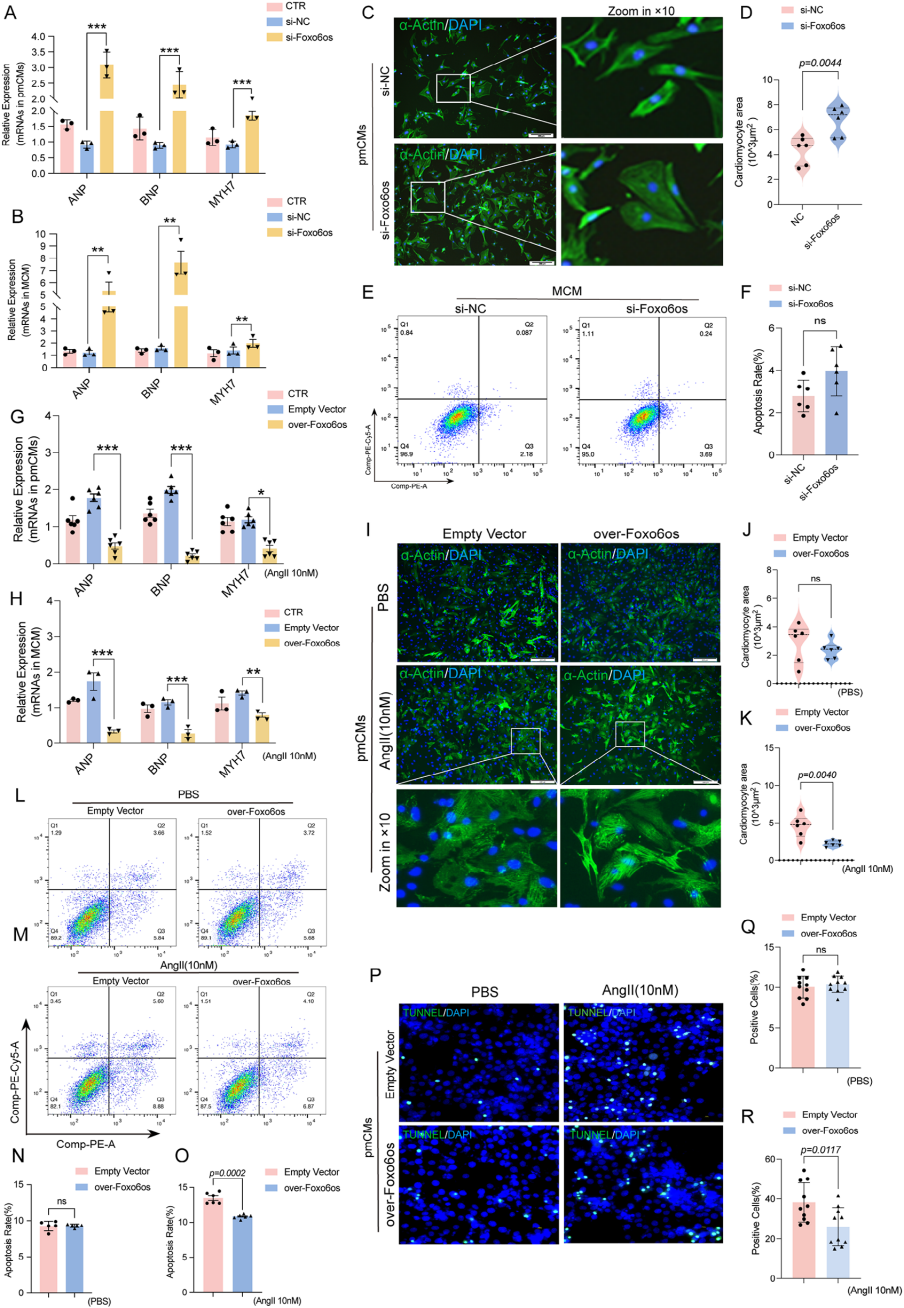
Foxo6os overexpression reduces Ang II‐induced cardiac hypertrophy in vitro. A,B) RT‐qPCR was performed to evaluate the expression levels of ANP, BNP, and MYH7 in pmCMs and MCM following a 48 h knockdown by si‐Foxo6os/NC (*n* = 3/group). C,D) Representative images of α‐actin/DAPI staining, as well as partial magnified views, and quantification of cardiomyocyte areas in pmCMs after transfecting with Foxo6os/NC siRNAs (*n* = 6/group). E,F) Flow cytometry analysis of apoptosis levels in MCM after treatment with si‐Foxo6os/NC for 48 h (*n* = 6/group). G,H) RT‐qPCR analysis of the expression levels of ANP, BNP, and MYH7 in both pmCMs and MCMs following 48 h of Ang II(10 nM) treatment, combined with Foxo6os overexpression (*n* = 6 in pmCMs group, *n* = 3 in MCM group). I–K) Representative image of α‐actin/DAPI staining, partial magnified views, and quantification of cardiomyocyte area in pmCMs after treatment with either PBS or Ang II(10 nM) for 48 h, combined with Foxo6os overexpression (*n* = 6/group). L–O) Representative flow cytometry analysis of apoptosis levels, as well as quantification of the apoptosis rate in MCM after treatment with either PBS or Ang II(10 nM) for 48 h, combined with Foxo6os overexpression (*n* = 5 in PBS group, *n* = 6 in Ang II group). P–R) TUNEL staining of pmCMs treated with either PBS or Ang II(10 nM) for 48 h, combined with Foxo6os overexpression, and corresponding quantification of positive cell numbers (*n* = 10/group). All experiments were performed with more than 3 independent replicates, ns = not significant, *p < 0.05, **p < 0.01, ***p < 0.001. These data are presented as means ± SD and analyzed using unpaired Student's *t*‐test.

To further investigate the functional role of Foxo6os in vitro, we transfected primary mouse cardiomyocytes (pmCMs) with three specific siRNAs targeting Foxo6os and selected si‐Foxo6os‐2 for subsequent experiments based on its optimal knockdown efficiency as determined by RT‐qPCR analysis (Figure S2A,B, Supporting Information). As expected, the inhibition of Foxo6os significantly increased the expression of ANP, BNP, and MYH7 (**Figure** **4**A,B), and accompanied with enlargement of cardiomyocyte sizes (Figure 4C,D). However, no significant apoptotic changes were observed between the si‐Foxo6os and si‐NC groups (Figure 4E,F). On the contrary, overexpression of Foxo6os (Figure S2C,D, Supporting Information) in Ang II‐treated cardiomyocytes could postpone the progression of cardiac hypertrophy, manifesting a significant reduction in the levels of ANP, BNP, and MYH7 compared to the control group (Figure 4G,H), and further confirmed by immunofluorescence (IF) staining (Figure 4I,J,K). Flow cytometry analysis also showed that forced‐expressing Foxo6os obviously reduced apoptosis in hypertrophic cardiomyocytes under Ang II treatment, compared to the PBS‐treated groups (Figure 4L,M,N,O). Moreover, TUNEL fluorescence staining verified that Foxo6os overexpression could downregulate the proportion of TUNEL‐positive cells in both Ang II‐treated cellular model (Figure 4P,Q,R through 4R) and the TAC‐induced mouse models (Figure S2E,F, Supporting Information). Taken together, Foxo6os is a crucial regulatory molecule in maintaining the internal stability of cardiac function.

### 2.5 Foxo6os Binds to MYBPC3 to Modulate Cardiac Remodeling

**Figure 5:**
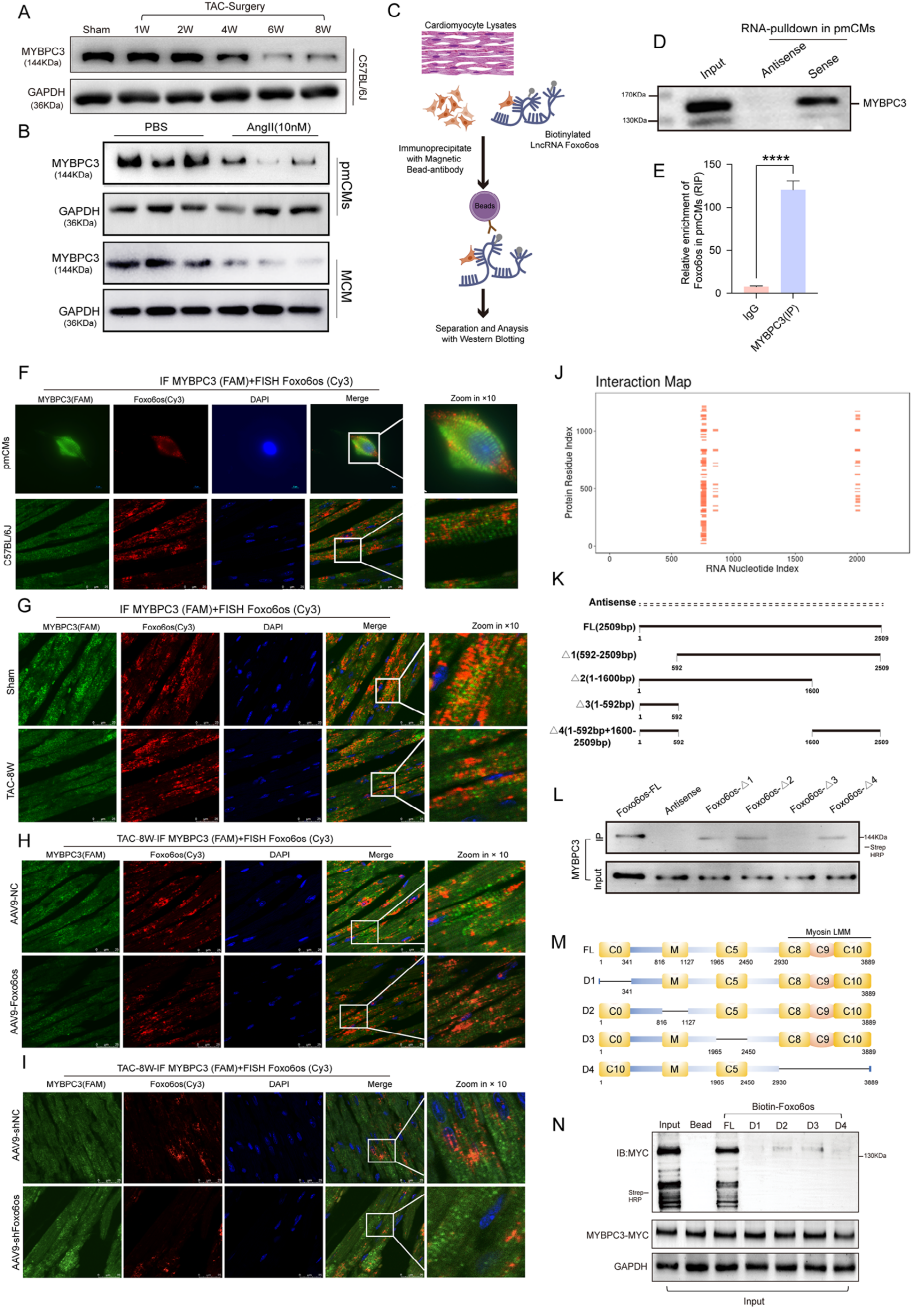
Foxo6os could directly bind to MYBPC3. A) Representative western blotting results of MYBPC3 respectively in sham and 1, 2, 4, 6, and 8 W post‐TAC surgery mice (*n* = 3/group). B) Representative western blotting results of MYBPC3 in pmCMs and MCM treatment with PBS/Ang II(10 nM) (*n* = 3/group). C) Schematic for biotin‐labeled RNA‐pulldown in pmCMs. D) Specificity of Foxo6os‐MYBPC3 interaction in biotin‐labeled RNA‐pulldown assay. E) RIP‐RT‐qPCR analysis of Foxo6os enrichment in MYBPC3 immunoprecipitated samples. F) Representative IF‐FISH images of Foxo6os (Cy3) and MYBPC3 (FAM) in pmCMs (*n* = 5) and C57BL/6J mice (*n* = 5). G) Representative IF‐FISH images of Foxo6os (Cy3) and MYBPC3 (FAM) in sham and TAC‐8 W mice (*n* = 5/group). H) Representative IF‐FISH images of Foxo6os (Cy3) and MYBPC3 (FAM) in AAV9‐NC and AAV9‐Foxo6os groups at 8 W post‐TAC surgery mice (*n* = 5/group). I) Representative IF‐FISH images of Foxo6os (Cy3) and MYBPC3 (FAM) in AAV9‐shNC and AAV9‐shFoxo6os groups at 8 W post‐TAC surgery mice (*n* = 5/group). J) Schematic representation of the predicted specific binding sites on Foxo6os for its interaction with MYBPC3. K) Illustration of the deletion mutations conducted on segments of Foxo6os. L) Biotin‐labeled RNA pull‐down assay identifying the specific binding sites on Foxo6os that interact with MYBPC3 using full‐length Foxo6os, and the deletion mutants Foxo6os‐△1 (592–2509 bp), Foxo6os‐△2 (1–1600 bp), Foxo6os‐△3 (1–592 bp), and Foxo6os‐△4 (1–592 bp + 1600–2509 bp). M) Schematic illustration of MYBPC3 structures and truncated mutants. N) Full‐length and MYBPC3 protein with a deletion mutation using a gene knockout expression vector were incubated with in vitro‐transcribed Foxo6os, subsequently pulled down by streptavidin beads. ****p < 0.0001. These data are presented as means ± SD and analyzed using unpaired Student's *t*‐test.

To elucidate the molecular mechanisms underlying Foxo6os‐mediated cardiac remodeling, we performed RNA sequencing on TAC‐induced hearts treated with either AAV9‐Foxo6os or AAV9‐NC. Comparative transcriptomic profiling revealed a cohort of mRNAs with distinct expression profiles in the AAV9‐Foxo6os‐treated group compared to the AAV9‐NC group. Gene enrichment analysis pinpointed that these mRNAs were associated with vascular smooth muscle contraction, DCM, and hypertrophic cardiomyopathy (HCM) (Figure S3A, Supporting Information). Notably, MYBPC3 was found to be significantly upregulated in response to Foxo6os upregulation (Figure S3B,D, Supporting Information). It's worth noting that the expression of MYBPC3 in TAC mice remained unchanged or even increased during the first 1–2 W post‐surgery. However, a slight downward trend in expression was observed at 4 W post‐surgery. After 6 W‐TAC surgery, the expression level dramatically decreased, which was nearly undetectable in 8 W‐TAC surgery mice (**Figure** **5**A; Figure S3C, Supporting Information). The expression pattern was recapitulated in Ang II‐induced cellular models of HF (Figure 5B; Figure S3E,F, Supporting Information). Moreover, Foxo6os overexpression in Ang II‐induced cardiomyocytes significantly elevated MYBPC3 mRNA levels, as illustrated in Figure S3G,H,I (Supporting Information), whereas the suppression of Foxo6os conversely reduced both MYBPC3 protein and mRNA transcript aboundance in cardiomyocytes (Figure S3J,K,L,M,N, Supporting Information). These results collectively indicated that Foxo6os regulates MYBPC3 expression through coordinated both transcriptional and post‐transcriptional mechanisms.

To further explore the interplay between Foxo6os and MYBPC3, we utilized two computational tools, LncLocator^[^ ^31^ ^]^ and DeepLncLoc^[^ ^32^ ^]^ to predict the subcellular localization of Foxo6os and found that Foxo6os primarily expresses in the cytoplasm, which notably coincides with the known localization pattern of MYBPC3 (Table S3, Figure S3O, Supporting Information). Then, by analyzing GeneFriends and an extensive RNA‐seq dataset of 112 heart tissue,^[^ ^33^ ^]^ a significant correlation (*p < 0.01*)  was revealed in possible between the transcriptional expression levels of Foxo6os and MYBPC3 (Figure S3P, Table S4, Supporting Information), which suggests that Foxo6os may interact with MYBPC3 in the regulation of HF progression.

Based on the bioinformatics references, we really confirmed the specific binding affinity of Foxo6os with MYBPC3 with RNA pull‐down assay (Figure 5C,D). RIP analysis also showed the specific interaction between MYBPC3 and Foxo6os (Figure 5E). IF‐FISH images revealed an overlap in the fluorescence signals of Foxo6os and MYBPC3 within mice cardiac tissues and isolated cardiomyocytes, indicating their co‐localization within the subcellular structure (Figure 5F). Besides, TAC‐8 W HF mice heart tissues displayed a decrease in the signal intensity of Foxo6os (Figure 5G). However, these phenomena were alleviated following AAV9‐Foxo6os treatment, as evidenced by the recovery of Foxo6os expression levels within the myofibrils and the restoration of myofibril morphology (Figure 5H). Conversely, Foxo6os knockdown exacerbated TAC‐induced myofibril disarray (Figure 5I). These results strongly implicating a potential role of Foxo6os in the regulation of MYBPC3 during cardiac remodeling. To identify the specific motif of Foxo6os for binding to MYBPC3, we conducted an in‐depth analysis of the results from catRAPID omics v2.0 to elucidate the potential binding sites^[^ ^12^, ^34^ ^]^ (Figure 5J), and then systematically truncated full‐length Foxo6os into fragments △1 (592–2509 bp), △2 (1–1600 bp), and △3 (1–592 bp), as well as the combined fragment △4 (1–592 + 1600–2509 bp) (Figure 5K). These four fragments were subjected to RNA pull‐down assay, respectively, which showed that the mutants Foxo6os‐△1, △3, and △4 were capable of binding to MYBPC3, similar to the full‐length of Foxo6os (Figure 5L). Subsequently, to systematically investigate the functional characteristics of Foxo6os domains binding to MYBPC3, we established an in vitro cardiomyocyte hypertrophy model with sustained Ang II stimulation for 48 h. As expected, endogenous Foxo6os expression was significantly downregulated under this pathological condition. However, transfection with either full‐length of Foxo6os or its mutants revealed that the cardiomyocyte cross‐sectional area in the Foxo6os‐△3 exhibited a typical pathological hypertrophy phenotype (Figure S3Q,R, Supporting Information). The mRNA levels of key hypertrophy markers (ANP, BNP, and MYH7) were also upregulated (Figure S4A, Supporting Information). Molecular docking simulations of Foxo6os‐cluster1(592–1600 bp) and Foxo6os‐cluster2 (1600–2509 bp) with MYBPC3 identified the spatial molecular structures of the binding sites interacting with MYBPC3 (Figure S4B,C, Supporting Information). The detailed docking regions are listed in Tables S5 and S6 (Supporting Information).

MYBPC3, a critical sarcomeric protein containing 10 functional domains, plays pivotal roles in cardiac structure and function. The N‐terminal (C0 domain) harbors a myosin‐binding domain, while the C‐terminal (C8–C10 domains) interact with titin, contributing to the stabilization of the sarcomere structure. Between the C1 and C2 domains lies a specific region, the M‐motif, which binds to the neck region of myosin and also serves as a phosphorylation site for MYBPC3.^[^ ^27^ ^]^ Notably, the C5 domain is unique to cardiac MYBPC3.^[^ ^22^, ^24^ ^]^ To further clarify the specific interaction regions between Foxo6os and MYBPC3, we constructed 4 different MYBPC3 deletion mutants and performed a biotin‐labeled pulldown assay (Figure 5M). As shown in Figure 5N, biotin‐labeled Foxo6os could enrich the M‐motif‐deletion and C5 domain‐deletion MYBPC3 mutant as efficiently as to the full‐length of MYBPC3. In contrast, the MYBPC3 mutants with C0 domain‐deletion and C8–C10 domain‐deletion exhibited no binding or weak binding with Foxo6os. The results demonstrated that both the N‐terminal (C0 domain) and the C‐terminal (C8–C10 domains) of MYBPC3 are essential for its interaction with Foxo6os. These structural domains may also play a crucial role in stabilizing Foxo6os and facilitating its involvement in the sarcomere, thereby contributing to myocardial contraction.

### 2.6 Downregulation of MYBPC3 Counteracts the Alleviative Effect of Foxo6os Overexpression on Cardiomyocyte Hypertrophy

**Figure 6:**
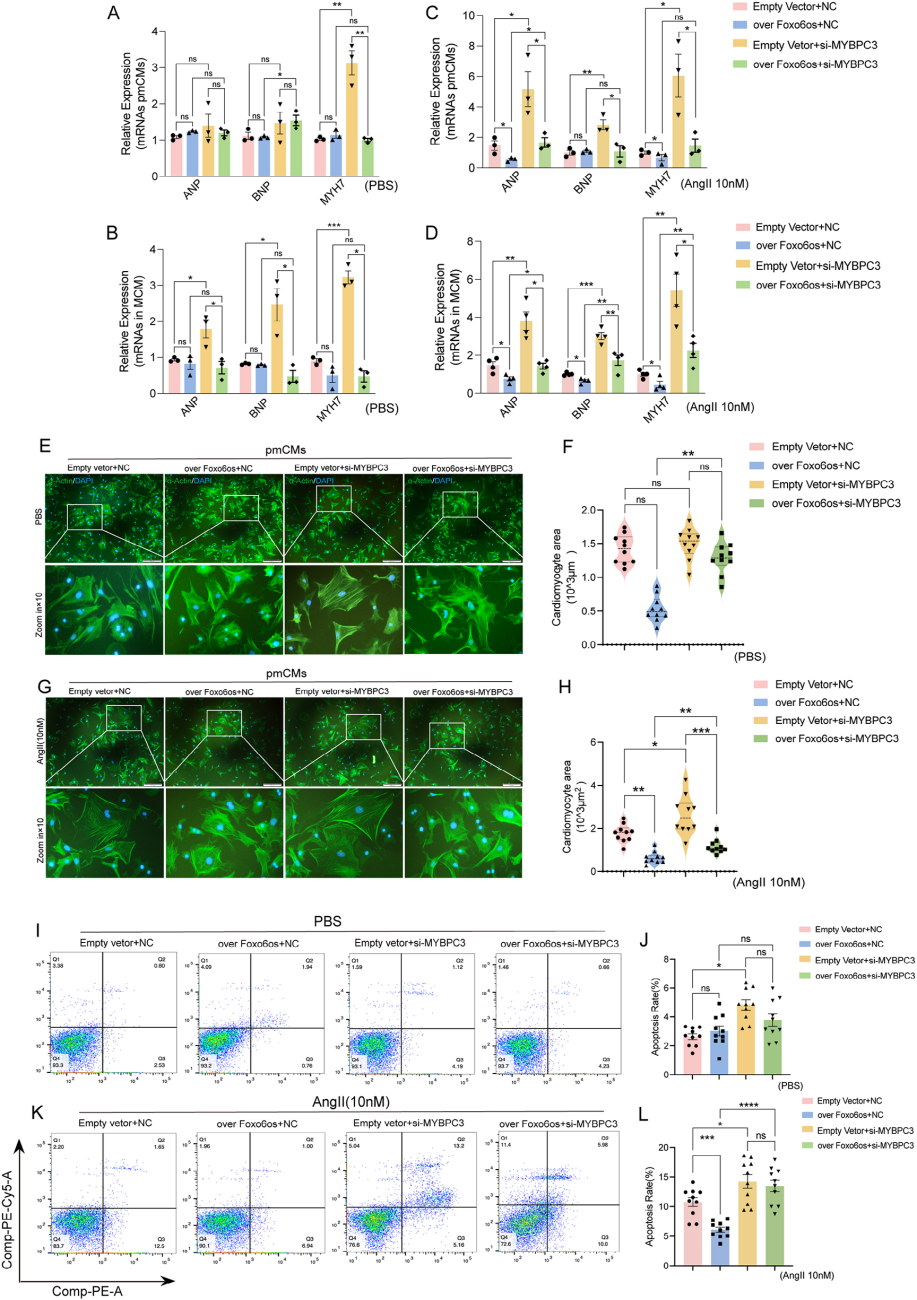
MYBPC3 downregulation offsets the protective effects of Foxo6os overexpression against cardiomyocyte hypertrophy. A,B) RT‐qPCR analysis of the mRNA expression levels of ANP, BNP, and MYH7 in pmCMs and MCM under PBS treatment for 48 h (*n* = 3/group). C,D) RT‐qPCR analysis of the mRNA expression levels of ANP, BNP, MYH7 respectively in pmCMs (*n *= 3) and MCM (*n* = 4) under Ang II (10 nm) treatment for 48 h. E,F) Representative α‐actin/DAPI staining, partial magnified views and the matching quantification of cardiomyocytes areas in pmCMs treated with PBS for 48 h (*n* = 10/group). G,H) Representative α‐actin/DAPI staining, partial magnified views and the matching quantification of cardiomyocytes areas in pmCMs treated with Ang II (10 nm) for 48 h (*n* = 10/group). I) Flow cytometry analysis of apoptosis levels in MCM treated with PBS for 48 h. J) Quantification of the data in I (*n* = 10/group). K) Flow cytometry analysis of apoptosis levels in MCM treated with Ang II (10 nM) for 48 h. L) Quantification of the data in K (*n* = 10/group). All experiments were performed with more than three independent replicates, ns = not significant, *p < 0.05, **p < 0.01, ***p < 0.001, ****p < 0.0001. These data are presented as means ± SD and analyzed using unpaired Student's *t*‐test.

To understand how MYBPC3 contributes its role in the regulation of Foxo6os for cardiac function, pmCMs, and MCM were first transfected with three si‐MYBPC3, respectively (Figure S5A, Supporting Information). Interestingly, the knockdown of MYBPC3 barely inhibited the effects of Foxo6os overexpression on cardiac remodeling in cardiomyocytes, showing with the stable levels of ANP, BNP, and MYH7 (**Figure** **6**A,B; Figure S5B,D, Supporting Information), as well as un‐changed cell sizes (Figure 6E,F). Also, apoptosis was not observed with huge differences under the condition (Figure 6I,J). Therefore, the regulatory effect of Foxo6os on MYBPC3 is relatively limited under normal conditions in cardiomyocytes.

In Ang II‐induced hypertrophic cardiomyocytes, however, inhibition of MYBPC3 distinctly reversed the effects induced by Foxo6os overexpression, such as upregulated the levels of ANP, BNP, and MYH7 (Figure 6C,D; Figure S5C,E, Supporting Information), enlarged cell areas of cardiomyocytes (Figure 6G,H), and accelerated the apoptotic process of cells (Figure 6K,L). Notely, the expression of MYBPC3 was sharply enhanced under Ang II‐induced myocardial remolding in both pmCMs and MCM when Foxo6os was overexpressed (Figure S5F,H,I, Supporting Information). In TAC‐8 W mice, the protein level of MYBPC3 was significantly increased in the AAV9‐Foxo6os treatment group compared to the control group, whereas it was partially reduced in the AAV9‐shFoxo6os treatment group (Figure S5G,J,K, Supporting Information). Overall, Foxo6os could interact with MYBPC3 to potentially mediate the process of cardiac hypertrophy.

### 2.7 Foxo6os Recruits PKC‐α to Phosphorylate MYBPC3 for Regulating Myocardial Contractility

It has been well established that the phosphorylation and dephosphorylation of MYBPC3 are mediated by a variety of kinases, including PKA and CaMK2δ.^[^ ^26^ ^]^ However, the role of PKC‐α in regulating myocardial contraction remains poorly understood.

**Figure 7:**
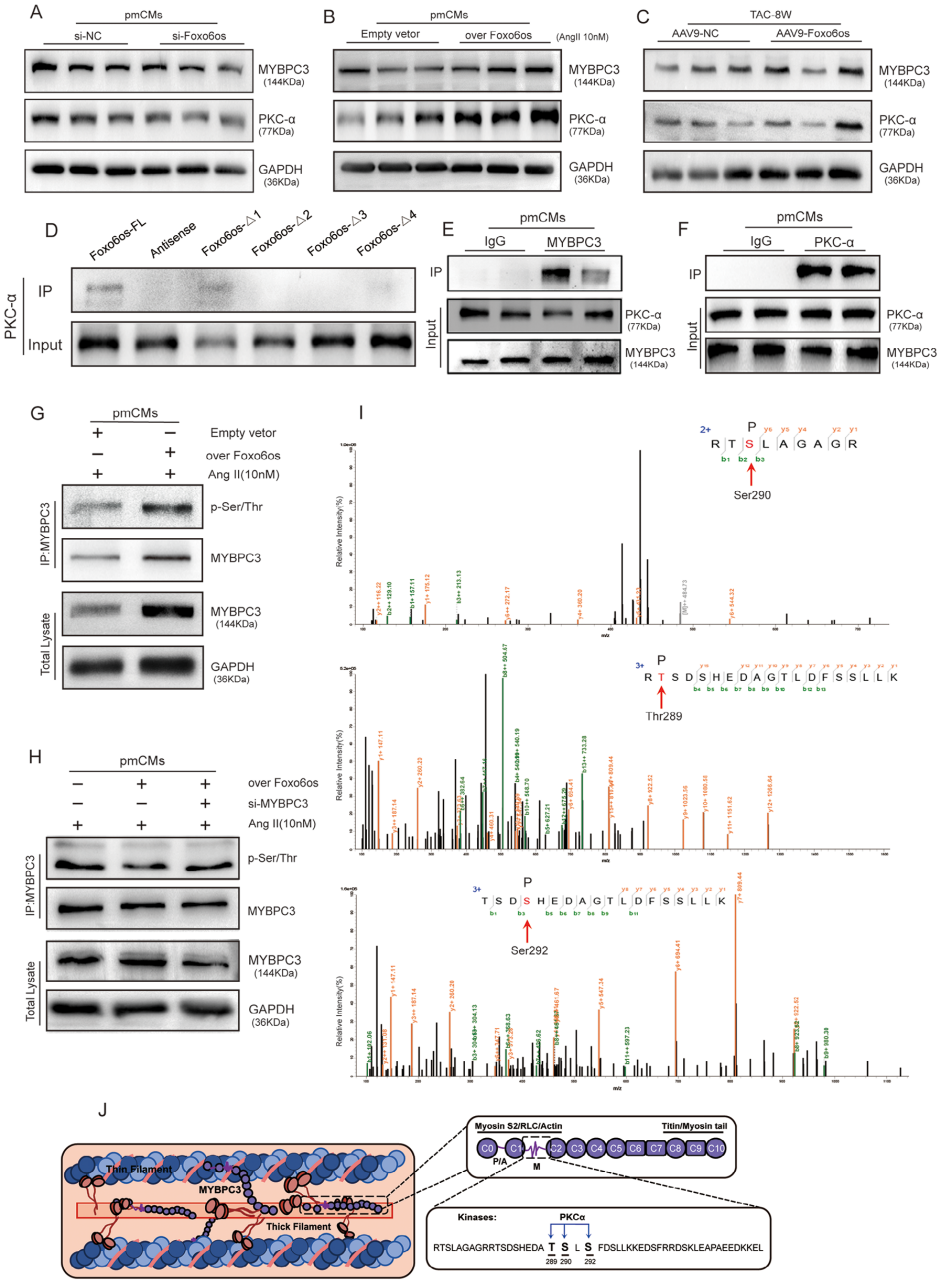
Foxo6os recruits PKC‐α to facilitate MYBPC3 phosphorylation at specific sites, forming a complex that regulates myocardial contractility. A) Western blot analysis of PKC‐α and MYBPC3 protein levels in pmCMs transfected with Foxo6os and NC siRNAs (*n* = 3/group). B) Western blot analysis of PKC‐α and MYBPC3 protein levels in pmCMs treated with Ang II (10 nM) for 48 h, following by Foxo6os expression (*n* = 3/group). C) Western blot analysis of PKC‐α and MYBPC3 protein levels in AAV9‐NC and AAV9‐Foxo6os groups at 8 W post‐TAC surgery (*n *= 5/group). D) RNA pull‐down assay identifying the specific binding sites on Foxo6os that interact with PKC‐α using full‐length Foxo6os, and the deletion mutants Foxo6os‐△1 (592–2509 bp), Foxo6os‐△2 (1–1600 bp), Foxo6os‐△3 (1–592 bp), and Foxo6os‐△4 (1–592 bp + 1600–2509 bp). E,F) Co‐IP assays in pmCMs were performed to enrich MYBPC3 using PKC‐α, and vice versa, followed by subsequent immunoblotting to detect their interactions (each group has two replicates). G,H) MYBPC3 was immunoprecipitated in cell lysis from Ang II (10 nM)‐induced pmCMs using MYBPC3‐specific antibody, and then phosphate Ser/Thr were detected. I) The LC/MS peak plot shows the fragmentation pattern of a phosphorylated peptide from MYBPC3. Phosphorylation at Ser290, Thr289 and Ser292 were indicated by a mass shift in the b‐ and y‐ion series, confirming the specific phosphorylation sites. J) Schematic for the structure and function of MYBPC3 within the sarcomere. MYBPC3 is composed of several Ig and FnIII domains, with a P/A linker and an m‐motif that contains multiple phosphorylation sites. Its C‐terminus integrates into the thick filament, while its N‐terminus can bind to both thin and thick filaments, regulating sarcomere contraction. The phosphorylation of the m‐motif between domains C1 and C2 modulates these interactions. Additionally, MYBPC3 may be phosphorylated by PKC‐α at Thr289, Ser290and Ser292. All experiments were performed with more than three independent replicates. These data are presented as means ± SD and analyzed using unpaired Student's *t*‐ test.

Next, we explored whether Foxo6os could regulate the phosphorylation of MYBPC3 through modulating PKC‐α. We found that both PKC‐α and MYBPC3 expression was downregulated in TAC‐8 W mice (Figure S6A,J,K, Supporting Information), and inhibition of Foxo6os in cardiomyocytes induced a decrease of PKC‐α and MYBPC3 expression in pmCMs (**Figure** **7**A; Figure S6D,E, Supporting Information) and MCM (Figure S6B,L,M, Supporting Information). Conversely, Foxo6os overexpression was associated with an increase of PKC‐α and MYBPC3 levels either in hypertrophic cardiomyocytes induced by Ang II (Figure 7B; Figure S6F,G,C,N,O, Supporting Information) or in TAC‐8 W mice treated with AAV9‐Foxo6os/NC (Figure 7C; Figure S6H,I, Supporting Information). Additionally, the upregulation of PKC‐α expression induced by the Foxo6os overexpression was partially reduced when co‐transfecting with si‐MYBPC3 (Figure S6P,Q, Supporting Information). Thence, Foxo6os could mediate MYBPC3 and PKC‐α levels in regulating the pathological development of HF.

Once more, we conducted biotin‐labeled RNA pull‐down assay to explore the detail of Foxo6os with PKC‐α and identified that Foxo6os could specifically enrich PKC‐α at the sites of 592–1600 and 1600–2509 bp, which coincide with the binding sites of Foxo6os and MYBPC3 (Figure 7D). Moreover, the endogenous co‐immunoprecipitation (co‐IP) assays also revealed an interaction between PKC‐α and MYBPC3 in cardiomyocytes, underscoring their mutual binding capacity (Figure 7E,F). Collectively, Foxo6os potentially serves as a “scaffold” to facilitate the interaction between PKC‐α and MYBPC3 in cardiomyocytes, resulting in the formation of a functional complex.

Given that PKC‐α acts as a kinase responsible for protein phosphorylation modifications, we further investigated whether Foxo6os could induce the activation of PKC‐α to phosphorylate MYBPC3 at a specific motif during HF. We overexpressed Foxo6os in Ang II‐induced pmCMs and subsequently enriched MYBPC3 from the cell lysate using magnetic beads. Immunoprecipitation was performed using antibodies against MYBPC3, and the serine/threonine (Ser/Thr) phosphorylation levels of MYBPC3 were determined using universal phospho‐Ser/Thr antibodies. Our results revealed that MYBPC3 displayed an increase level of Ser/Thr phosphorylation upon overexpression of Foxo6os in the Ang II‐induced cardiomyocyte hypertrophy model (Figure 7G), but the alternation of Ser/Thr phosphorylation was blocked upon simultaneous MYBPC3 knockdown (Figure 7H). We next employed biotin‐labeled Foxo6os for RNA pull‐down assay in pmCMs to capture MYBPC3 and detected MYBPC3 specific sites using liquid chromatography‐tandem mass spectrometry (LC‐MS) (Figure S6R, Supporting Information). The results displayed that the phosphorylation sites identified in the enriched MYBPC3 were located at Thr289, Ser290, and Ser292 (Figure 7I; Figure S6S, Supporting Information), which differ from the previously reported phosphorylation sites of MYBPC3 in its m‐motif. We thus supposed that Foxo6os could recruit PKC‐α to facilitate the phosphorylation of MYBPC3 at the specific motifs, which are biologically plausible sites that may be independently regulated by kinases of PKC‐α (Figure 7J).

### 2.8 Foxo6os‐MYBPC3 Enhances Myocardial Contractility Upon L‐Type Calcium Channel

**Figure 8:**
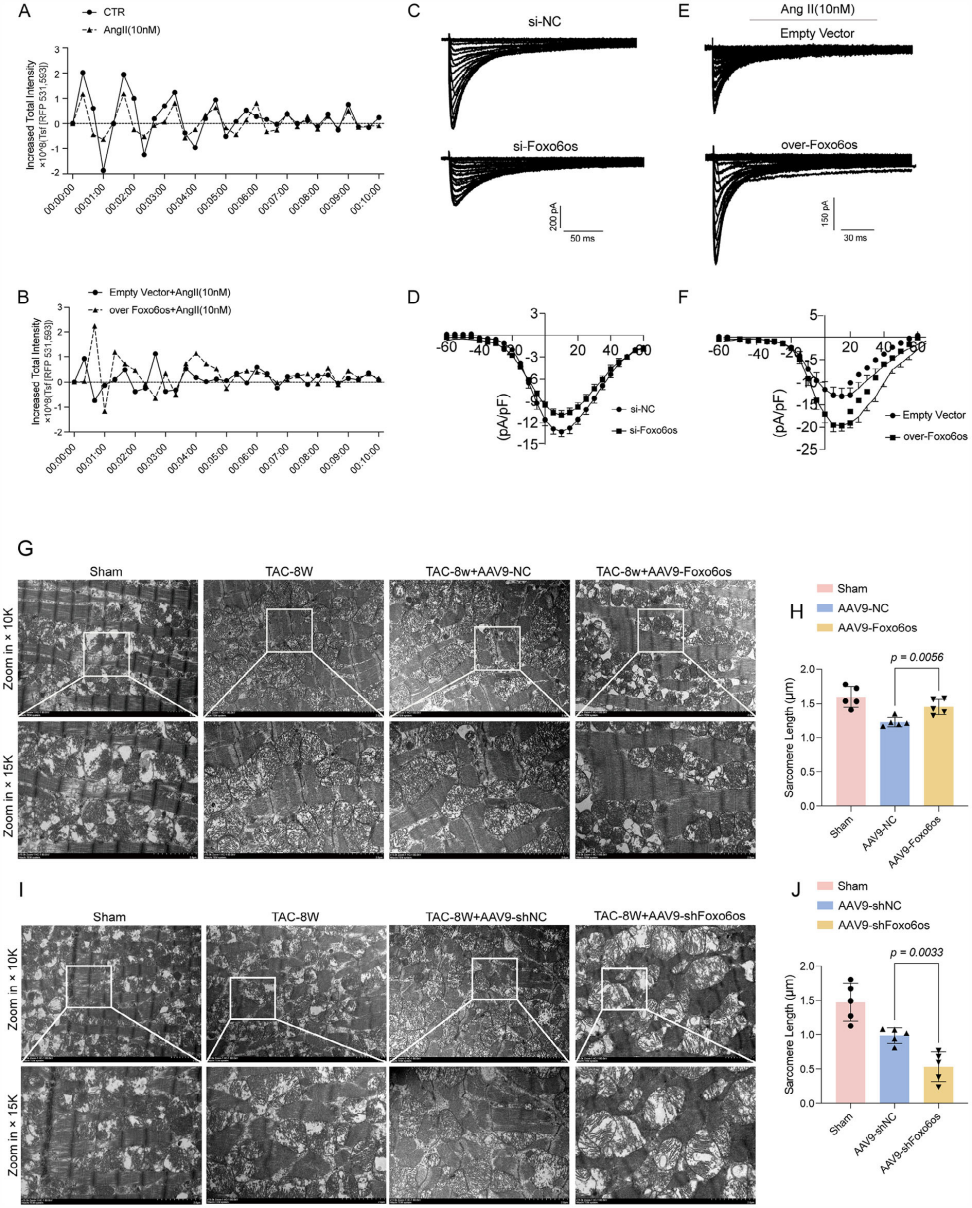
Foxo6os‐MYBPC3 Interaction Enhances Myocardial Contractility Through LTCC Dependency. A,B) Changes in the average transient fluorescence intensity of calcium ions induced by spontaneous contractions of cardiomyocytes (*n* = 5/group). C,D) Whole‐cell patch clamp analysis of isolated cardiomyocytes to measure *I*_CaL_ in si‐NC and si‐Foxo6os groups (*n* = 3/group). E,F) Whole‐cell patch clamp analysis of isolated cardiomyocytes to measure I_CaL_ in empty vector and Foxo6os overexpression groups treated with Ang II (10 nM) for 48 h (*n* = 3/group). G) Representative TEM images showing changes in sarcomere morphology in the sham, TAC‐8 W, and AAV9‐NC/Foxo6os groups (*n* = 5/group). H) Statistical analysis of sarcomeres length in G (*n* = 5/group). I) Representative images of sarcomere morphology changes observed by TEM in the sham, TAC‐8 W, and AAV9‐shNC/Foxo6os groups (*n* = 5/group). J) Statistical analysis of sarcomeres length in I (*n* = 5/group). All experiments were performed with more than three independent replicates. *P *< 0.05 indicates statistical significance. These data are presented as means ± SD and analyzed using unpaired Student's *t*‐test.

Given that MYBPC3 phosphorylation plays a critical role in regulating cardiac myofilament function, the next question is whether the effects of Ang II‐induced effects on cardiomyocyte Ca^2+^ handling are modulated by Foxo6os overexpression. We first found that Ca^2+^ transient properties were inhibited under the condition of Ang II‐induced decompensated hypertrophy (**Figure** **8**A, Videos S1 and S2, Supporting Information), but this phenomenon could be alleviated in the presence of Foxo6os overexpression (Figure 8B, Videos S3 and S4, Supporting Information). Unfortunately, because pmCMs were unable to maintain viability under the strong illumination of a confocal microscope's laser source, we only detected the fluorescence intensity changes of Ca^2+^ within 10s. Previous evidence indicates that Ang II‐induction could facilitate L‐type calcium channel (LTCC) internalization via β‐arrestin1 recruitment, resulting in decreased LTCC in cardiomyocytes, potentially affecting their contractility.^[^ ^35^ ^]^ Moreover, the phosphorylation of MYBPC3 is part of the β‐adrenergic signaling cascade, which also regulates LTCC.^[^ ^36^ ^]^ Therefore, we tested whether the enhanced contractile function following overexpression of Foxo6os in Ang II‐induced pathological hypertrophy is related to the L‐type Ca^2+^ current (l_CaL_). A whole cell patch clamp from isolated cardiomyocytes was used to measure I_CaL_. We found that Foxo6os knockdown may result in a marked decreased in *I*_CaL_ and an upward of the *I–V* curve, indicating a lower activation voltage threshold and compromised excitability or contractility of cardiomyocytes (Figure 8C,D). Moreover, the Ang II‐induced stimulation of *I*_CaL_ was almost fully promoted in the presence of Foxo6os overexpressing (Figure 8E,F). In addition, transmission electron microscopy (TEM) revealed that the TAC‐8 W mice displayed a decrease of the number of mitochondria in the left ventricular myocardium with messy distribution, asymmetric sarcomere, and blurred structure of the Z‐lines and H‐lines. Moreover, Foxo6os overexpression in TAC‐8 W mice improved sarcomere length with a more organized myofibril structure, though some sarcomere areas were twisted and misplaced, an overall increase in length was detected (Figure 8G,H). However, in TAC‐8 W mice treated with AAV9‐shFoxo6os/NC, the myocardial myofibrils were severely twisted and widely misaligned, with a significant shortening of the sarcomere length. Additionally, the myofibrillar structure became disorganized and disrupted, ultimately leading to a marked reduction in overall length (Figure 8I,J). These findings suggest that Foxo6os may regulate myocardial contraction by modulating the phosphorylation status of MYBPC3, which in turn affects downstream Ca^2+^ levels, thereby exerting a positive regulatory effect on cardiac contractility.

### 2.9 Human‐Derived LncRNAs Likely Mirrors Foxo6os Function in HF

To clarify whether the human genome contains the lncRNAs with functional similarity to Foxo6os that play analogous roles in HF, we employed seekr,^[^ ^37^ ^]^ a computational tool that evaluates sequence similarity by calculating Pearson's correlation, to measure the similarity between the k‐mer profiles of human lncRNAs and the Foxo6os. By calculating and converting the k‐mer profiles into *z*‐scores, we identified 1505 lncRNA transcripts with *z*‐scores greater than 2 (Table S7, Supporting Information). To further ensure the consistency between the functions of the selected human lncRNAs and Foxo6os, we analyzed the RNA‐seq dataset (GSE141910), and t‐SNE dimensionality reduction effectively distinguished samples from healthy and HF human left ventricles (Figure S7A, Supporting Information). Subsequently, we identified significantly downregulated lncRNAs between HF patients and healthy controls, among which 7 lncRNAs had seekr correlation coefficient‐transformed *z*‐scores greater than 2 (Figure S7B,C, Table S8, Supporting Information). Finally, based on the annotations linking these lncRNAs to cardiac diseases and functions, we selected 5 lncRNAs for further experimental studies.

To validate the correlation between the 5 lncRNAs and MYBPC3 in human samples, we explored RNA pull‐down assay in human embryonic stem cells‐derived cardiomyocytes (hESCs‐CM). The results indicated that lncRNA AL021392.1, AC090921.1, and ELF3‐AS1 could interact with potentially enriched MYBPC3 (Figure S7D, Supporting Information). Additionally, we extracted exosomal RNA from the peripheral blood of clinical HF patients and analyzed the plasma expression levels of these lncRNAs, comparing them to those in healthy controls. The RT‐qPCR analysis demonstrated a significant downregulation of AL021392.1 in HF patients, while the expression levels of AC090921.1 and ELF3‐AS1 showed an upward trend, but without statistical significance (Figure S7E, Supporting Information). These findings suggest that AL021392.1 exhibits a downregulation trend in HF patients, consistent with the regulatory pattern of Foxo6os, implying its potential as a serum biomarker for the diagnosis of HF patients.

In summary, this study confirms that Foxo6os serves as a “scaffold”, linking MYBPC3 and PKC‐α to form a complex that regulates the biological processes of myocardial contraction during the pathophysiological process of HF, which may provide potential biological targets for the diagnosis and therapeutic intervention in clinical HF patients (Figure S7F, Supporting Information).

## 3 Discussion

In our study, we identified a novel lncRNA Foxo6os, which exhibits high cardiac specificity and is significantly downregulated in a murine model of HF. This finding underscores the potential role of Foxo6os in the pathogenesis of HF. Indeed, forced‐expressing Foxo6os could enhance cardiac function and protect against adverse cardiac remodeling during hypertrophy and HF. Reversely, Foxo6os knockdown could impair cardiac function and exacerbate adverse cardiac remodeling during cardiac hypertrophy and HF. Upon overexpression of Foxo6os in an Ang II‐induced hypertrophic cardiomyocyte models, we found a distinct reduction in the expression of hypertrophy‐related markers, along with a notable decrease in both cardiomyocyte sizes and apoptosis levels, demonstrating, for the first time, the significant role of Foxo6os in maintaining cardiac homeostasis.

Recent evidences have indicated that Foxo6, Foxo6os's host gene, is involved in both neurodegenerative and cardiovascular diseases,^[^ ^38^ ^]^ for example, Foxo6 could influence cardiac remodeling following myocardial infarction (MI) by promoting cardiomyocyte apoptosis.^[^ ^39^ ^]^ Also, Foxo6 plays a protective role for the heart under metabolic stress by enhancing the expression of genes related to fatty acid oxidation.^[^ ^40^ ^]^ On the other hand, Foxo6os, as a translational strand, can modulate the transcription and stability of the Foxo6 gene via forming double‐stranded structures with its corresponding mRNA, thereby influencing its expression.^[^ ^41^ ^]^ Given that Foxo6 has been gradually recognized as a potential therapeutic target for heart disease, it is necessary to concern the synergistic effects between Foxo6 and Foxo6os when developing specific molecular targeted against Foxo6. Despite Foxo6os overexpression or knockdown achieved by the AAV9‐cTnT system in mice with myocardial injury could effectively influence cardiac dysfunction, however, there still exist certain limitations, such as individual differences among animals and partial clearance by the liver after entering through the portal vein, leading to poor stability. Therefore, further experiments should conduct cardiomyocyte‐specific Foxo6os knockout mice using the *cre‐loxP* system to explore whether deleting Foxo6os during embryonic development could lead to HF.^[^ ^42^ ^]^

The importance of lncRNA‐protein interactions has been recently elucidated during the development of cardiovascular diseases.^[^ ^43^ ^]^ Here, we identified a promising lncRNA‐protein partner, Foxo6os‐MYBPC3, and confirmed their interaction in hypertrophy models. MYBPC3, a sarcomeric protein, anchors to the thick filament via its C‐terminal domain. By competitively inhibiting the interaction between myosin heads and actin, the N‐terminal region of MYBPC3 is capable of binding to actin. This interaction modulates the ATPase activity of myosin, thus regulating the rate of muscle contraction.^[^ ^44^ ^]^ During the relaxation phase of cardiac contraction, phosphorylation of MYBPC3 ensures the dissociation of myosin heads from actin, then facilitating the sliding of the actin filaments.^[^ ^29^ ^]^ The M‐domain of MYBPC3 is a key structure located in its C‐terminal region, which commonly referred as the C8‐C10 segment,^[^ ^45^ ^]^ which contains important binding sites for interacting with myosin. Particularly, the C10 domain in the C‐terminal region is responsible for directly binding to the thick filaments (myosin), thereby helping to position and stabilize the function of the cardiac sarcomere. As it is among the most common genetic mutations contributing to HCM, mutants of MYBPC3 are a crucial players in HCM patients.^[^ ^46^, ^47^ ^]^ Mutations in the MYBPC3 gene may lead to changes in the amino acid sequence, which affects the 3D structure of MYBPC3.^[^ ^24^ ^]^ These mutations disrupt its interaction with actin or myosin, destabilize the protein's stable structural domains, along with increased the instability and degradation of MYBPC3.^[^ ^44^ ^]^ In certain cases, mutations may confer new functions upon MYBPC3 or abnormally enhance its existing functions, in which results in excessive contraction of the sarcomere.

In this study, we observed a downregulation of MYBPC3 in HF mouse models. In the early stages of myocardial hypertrophy, MYBPC3 expression undergoes compensatory upregulation to maintain cardiac function. However, as the disease progresses, stress‐induced dysregulation of MYBPC3 expression exacerbates myocardial fiber dysfunction, ultimately contributing to the development of HF. But it remains to be determined whether this reduction is due to mutations that cause an increase of protein instability and degradation. Besides, Foxo6os could interact with MYBPC3 both on its N‐terminal and the C‐terminal and upregulate its expression under Ang II‐induced myocardial injury and cardiac dysfunction. We hypothesize that Foxo6os and MYBPC3 interact with each other and localize at opposite ends of the sarcomere, near myosin and actin filaments, respectively. This interaction likely facilitates the cooperative stabilization of MYBPC3 within the sarcomere. Conversely, the presence of MYBPC3 may contribute to stabilizing Foxo6os in the sarcomere, enabling its precise involvement in the regulation of sarcomeric contraction.

In addition, MYBPC3 is highly phosphorylated under basal conditions, but the modification is significantly inhibited in cardiac diseases.^[^ ^27^ ^]^ The phosphorylation status of MYBPC3 is involved in the regulation of heart contractility through its interaction with thick and thin filaments. In cardiomyocytes, PKA is the well‐established activator in the β‐adrenergic receptor pathway, leading to the phosphorylation of MYBPC3 at specific sites, including Ser273, Ser282, and Ser302.^[^ ^28^ ^]^ CaMKII is another enzyme that phosphorylates MYBPC3, typically associated with calcium signaling as a player in regulating cardiac function.^[^ ^48^ ^]^ In this work, we demonstrated that Foxo6os can recruit protein kinase PKC‐α, which in turn mediates phosphorylation of MYBPC3 at the N‐terminal region (Thr289, Ser290, Ser292) during cardiac hypertrophy. Foxo6os functions as a molecular “scaffold”, facilitating the assembly of a ternary complex comprising MYBPC3 and PKC‐α, which is essential for the maintenance of normal myocardial contractile function. Although we have specifically examined the phosphorylation sites of MYBPC3 targeted by Foxo6os, a more detail analysis by point mutation at the three modified sites needs to be further explored to confirm whether PKC‐α can still associate with MYBPC3. Our study has revealed the pivotal association between Foxo6os‐mediated myocardial contraction and the phosphorylation status of MYBPC3, suggesting that it is imperative to contemplate the synergistic actions of Foxo6os and PKC‐α to fully harness the therapeutic potential of this pathway to develop novel small molecule drugs for the intervention of HF.

Calcium signaling is much crucial in the process of myocardial contraction^[^ ^25^ ^]^ that usually starts with the propagation of an action potential (AP). When the membrane of the cardiomyocyte depolarizes, voltage‐gated LTCC will open to allow the amount of Ca^2^⁺ flow into the cells. This influx of Ca^2^⁺ triggers the opening of ryanodine receptors (RyR2) in the sarcoplasmic reticulum (SR), leading to a large release of Ca^2^⁺ into the cytoplasm.^[^ ^49^ ^]^ The increased cytoplasmic Ca^2^⁺ concentration interacts with troponin C (TnC) within the sarcomere, inducing a conformational change in the troponin complex, in which the shift moves tropomyosin away from actin's binding sites, thereby exposing the myosin binding sites on actin.^[^ ^36^ ^]^ The phosphorylation of MYBPC3 in response to calcium signaling relieves its inhibitory effects on myosin, allowing more efficient binding to actin filaments and facilitating stronger and more coordinated sarcomere contraction.^[^ ^26^ ^]^ We here found that Foxo6os knockdown reduced L‐type calcium current amplitude, leading to decreased Ca^2^⁺ influx and impaired cardiac contraction. Conversely, forced expression of Foxo6os can effectively activate LTCC in cardiomyocytes, leading to a significant increase in the influx of calcium ions. This enhancement in calcium flow provides a certain degree of recovery potential for the impairment of myocardial contractile function during the process of HF. Therefore, the effect of Foxo6os on gating L‐type calcium current is valuable for clinical application in the treatment of HF. Its impact on this current offers a promising therapeutic target for HF treatment, potentially improving calcium handling and cardiac function.

Many lncRNAs have been revealed to module the process of cardiovascular diseases. For instance, the levels of lncRNA nuclear paraspeckle assembly transcript 1 (NEAT1) and matrix metalloproteinase‐9 (MMP‐9) in the serum exosomes of patients with ST‐segment elevation myocardial infarction (STEMI) are notably higher than in patients with unstable angina or non‐MI.^[^ ^50^ ^]^ Thus, the two lncRNAs has been suggested as independent predictors of STEMI by logistic regression analysis. Additionally, lncRNA ENST00000556899.1 and ENST00000575985.1 were upregulated in the plasma exosomes of acute myocardial infarction (AMI) patients,^[^ ^51^ ^]^ which are also associated with clinical processes such as inflammatory and myocardial injury prognosis, suggesting their potential in reflecting the severity of AMI and predicting patient outcomes.^[^ ^52^ ^]^ We also identified several lncRNAs that exhibit functional similarity with Foxo6os in human HF patients. Among the candidates, 3 lncRNAs show a possible role in interacting with MYBPC3. Notably, lncRNA AL021392.1 exhibits a significant downregulation trend in the peripheral blood of HF patients, which is consistent with the expression pattern of Foxo6os in the cardiac tissue of HF mice. Moreover, whether AL021392.1 could play a similar function to Foxo6os during the progression of HF patients remains to be further investigated. Moreover, the lncRNAs in patient plasma exhibit dynamic changes, and whether such changes can serve as reliable indicators for the early diagnosis of HF, requires rigorous validation through standardized and comprehensive studies. These studies must take into account the variability across different populations and stages of disease progression to ensure clinical applicability and reliability.

In conclusion, Foxo6os exhibited a downregulation along with the progression of hypertrophy, and overexpressing the lncRNA largely alleviated the phenotype of cardiac remolding. Foxo6os as a “scaffold” not only associates directly with MYBPC3, also recruits PKC‐α, which mediates phosphorylation of MYBPC3 at specific sites. This post‐translational modification is crucial for maintaining cardiac contraction by gating LTCC, especially in the pathological conditions caused by myocardial hypertrophy. These results provide novel insights into the molecular regulation following myocardial injury, providing a valuable reference for developing therapeutic strategies targeting HF.

## 4 Experimental Section

### Animal Model and Treatment

Male C57BL/6J mice aged 6–7 W were purchased from the JieSiJie Laboratory Animal Co., Ltd. (Shanghai), and maintained in a pathogen‐free environment for 2 W with adequate access to food and water. All experimental procedures received approval from the Ethics Committees of Tongji University School of Medicine, and subsequently complied with the Guide for the Care and Use of Laboratory Animals by the National Institutes of Health.

Given that estrogen, along with diminished levels of testosterone, might bestow a shield against cardiac fibrosis,^[^ ^53^, ^54^ ^]^ male mice were only selected for animal experiments in the present studies. Consequently, all animal experiments were carried out using male mice to provoke robust tissue failure and cardiac fibrosis.

### Transverse Aortic Constriction (TAC)‐Induced Cardiac Hypertrophy Models

Regarding to the cardiac hypertrophy model, the TAC was conducted through the implementation of a transverse incision in the chest of mice using a thoracotomy technique. The mice were anesthetized with 1.5% isoflurane inhalation, mechanically ventilated, and positioned supine on a heated surgical table. The ventilator settings were adjusted to achieve a respiratory ratio of 1:1.5, a respiratory rate of 140 breaths per minute, and an inspiratory volume of 1 mL. For enhanced visualization, a high‐power cold light source was positioned in close proximity to the mouse's neck, and surgical forceps were utilized to expose the glottis by retracting the tongue root. Under direct vision, a 22 G intravenous catheter without the needle tip was inserted into the mouse's trachea via the tracheal intubation needle; successful intubation was confirmed by observing consistent chest wall movement with ventilator frequency. Utilizing an open‐chest approach under the magnification of a dissecting microscope, an incision was made along with a horizontal section of the second rib followed by dissection of the thymus for exposure of the aortic arch region. The chest retraction technique was employed for stabilizing the sternum while ensuring sufficient surgical workspace. Identification of the aortic arch following by passing 6‐0 suture horizontally through the brachiocephalic trunk and left subclavian artery using the guidewire technique; a loose knot was secured in the aorta before positioning the 27G blunt needle parallel to it. A square knot was swiftly fastened at the tip of the needle prior to withdrawal, resulting in the formation of a 0.4 mm constriction. The chest retractor was carefully removed and evacuated any excess gas from the thoracic cavity. The sternum and ribs were closed using interrupted 6‐0 Prolene sutures, followed by continuous closure of the skin with 4‐0 Prolene sutures. Mechanical ventilation was temporarily paused and uncovered the cannula. Once spontaneous breathing resumed in the mouse, it was transferred to a 37 °C heating pad and allowed ≈20 mins for recovery of consciousness. Subsequently returned it to its cage, marking the completion of the surgical procedure.

### Treatment of Adeno‐Associated Virus Serotype 9 (AAV9)

The AAV9 viruses were obtained from Genomeditech Technology (Shanghai, China). These viruses, which carry the Foxo6os coding sequence under the control of a cTnT promoter (AAV9‐cTnT‐Foxo6os/AAV9‐cTnT‐shFoxo6os), or the control virus (AAV9‐cTnT‐NC/ AAV9‐cTnT‐shNC), were injected into the tail vein of 8 W‐old C57BL/6J mice at a dose of 5 × 10^12 ^VG.

### Echocardiography

Cardiac function was evaluated by the multi‐mode small animal ultrasound imaging system (Vevo 3100, FUJIFILM Visual Sonics, Canada). Mice were anesthetized with isoflurane (1.5%) in oxygen (1 L min^−1^) and subsequently restrained on a 37 °C plate without mechanical ventilation to avoid bradycardia. Indices of murine cardiac short‐axis sections, aortic blood flow velocity, were obtained to evaluate the extent of ligation. M‐mode ultrasound images of the left ventricular long axis were obtained to assess left ventricular function. Parameters including heart rate, end diastolic interventricular septum thickness (IVS;d), end systolic interventricular septum thickness (IVS;s), left ventricular end diastolic diameter (LVID; d), left ventricular end systolic diameter (LVID; s), left ventricular end‐diastolic posterior wall thickness (LVPW; d), left ventricular end‐systolic posterior wall thickness (LVPW; s), LVEF and FS, LVEDV and LVESV were gathered for further analysis.

### Cell Cultures and Transfection

MCM (mouse cardiomyocytes) were purchased from the Cell Bank of Type Culture Collection of the Chinese Academy of Sciences. Detailed information on MCM can be found in Material S1 (Supporting Information). MCM were cultured in DMEM (Gibco, Thermo Fisher Scientific, Waltham, MA, USA), supplemented with 10% fetal bovine serum (FBS, Gibco; Thermo Fisher Scientific, Inc.) and 1% penicillin‐streptomycin (Gibco, Thermo Fisher Scientific, Waltham, MA, USA) at 37 °C in a humidified atmosphere containing 5% CO2. Regarding to the transfection, the cells planted in a 6‐plate well. When the density had reached 50%‐70%, 5 µg plasmids were transfected in each well by adding Xfect Transfection Reagant (#631317, Takara Bio Inc., Shiga, Japan) of 4.5 µL. Similarly, 100 pmol siRNA (RiboBio Co., Ltd., Guangzhou, China) were transfected by using Xfect RNA Transfection Reagant of 10 µL (#631450, Takara Bio Inc., Shiga, Japan) in each well. The transfected cells were cultured at least 48 h later for subsequent experiments. The sequences of siRNAs are listed in Table S2 (Supporting Information).

### Cardiomyocyte Isolation and Treatments

For the isolation of pmCMs from postnatal mouse hearts, C57BL/6J 1‐day‐old neonatal mice were purchased from the JieSiJie Laboratory Animal Co.,Ltd. (Shanghai). Briefly, the fresh hearts were immersed in ice‐cold PBS and promptly diced into 1mm^3^ fragments, following by incubating with 0.1% trypsin (Gibco, Thermo Fisher Scientific, Waltham, MA, USA) at 4 °C overnight. After incubation, the tissues were digested with DMEM containing 10% FBS and 0.1% collagenase type 2 (Yeasen Biotechnology Co., Ltd., Shanghai) for 7 mins at 37 °C with agitation. The digested solution was transferred to a new tube and replaced with fresh buffer for another 2 times. Then the dispersed cells were filtered with a 70 µm cell sieve. For myocardial cell purification, the filtered cell suspension was transferred to a culture dish and incubated at 37 °C for 60 mins. Non‐myocardial cells, with strong adhesion properties, adhered to the dish surface, while myocardial cells, due to weaker adhesion, remained suspended. After incubation, the myocardial cell‐enriched suspension was collected, and non‐adherent cells were discarded. The cells were then cultured in a medium containing 0.1 mm BrdU and 1% VB_12_, for further experiments. The unattached viable cells, rich in cardiomyocytes, were collected to culture on plates in DMEM supplemented with 10% FBS.

### Angiotensin II (Ang II)‐induced Cardiac Hypertrophy Cell Model

To model the decompensated pathological state of cardiac hypertrophy in vitro, MCM and pmCMs were treated with Ang II (10 nM, #HY‐13948, MedChemExpress) for 48 h.

### RNA Extraction and Real‐time Quantitative Polymerase Chain Reaction (RT‐qPCR)

The total RNA was isolated from cardiac tissues and cultured cells utilizing Trizol reagent (Vazyme Biotech Co., Ltd. Nanjing, China) according to the manufacturer's protocol. Subsequently, reverse transcription was performed with 1 µg of purified RNA using a Takara PrimeScriptTM RT reagent kit with a gDNA eraser (#RR037A, Takara Bio Inc., Shiga, Japan). The quantitative PCR reactions were performed in duplicate using cDNA and ChamQ SYBR qPCR Master Mix (Vazyme Biotech Co., Ltd. Nanjing, China) on the CFX96 Real‐Time PCR Detection System (Bio‐Rad Laboratories, Hercules, CA, USA) under standard manufacturer's conditions. The results were analyzed through the approach of 2^−ΔΔCt^ to determine fold change compared to the control sample, with normalization against the GAPDH. The primers used are listed in Table S1 (Supporting Information).

### Western Blotting and Coimmunoprecipitation (Co‐IP)

The proteins were extracted from homogenized mouse myocardial tissue, as well as lysed cardiomyocytes in RIPA solution (#P0013B, Beyotime Biotechnology, Shanghai, China,), with protease/phosphatase inhibitor cocktail (#PPC1010, Sigma Aldrich, USA). The concentration was quantitated using Pierce BCA Protein Assay Reagent (#23227, Thermo Fisher Scientific, Waltham, USA). Subsequently, total proteins (10–20 µg) were separated by sodium dodecyl sulfate‐polyacrylamide gel electrophoresis (SDS‐PAGE) and then transferred to polyvinylidene difluoride (PVDF) membranes (Millipore, MA, USA). The membranes were blocked with 5% BSA (#36101ES25, Yeasen Biotechnology Co., Ltd., Shanghai, China,) for 2 h, after which they were incubated with primary antibodies at 4 °C overnight. Following 3 washes with TBST, the membranes were incubated with HRP‐conjugated secondary antibodies (#SA00012‐1 1:10000, Proteintech, Wuhan, China). Ultimately, the protein bands were visualized by employing the ECL Regaent (Epizyme Biotechnology Co., LTD, Shanghai). The primary antibodies used in western blotting are as follows: GAPDH (1:5000, #60004; Proteintech, Wuhan, China); MYBPC3 (1:1000, #sc‐137237; Santa Cruz, CA, USA); PKC‐α (1:1000, #21991‐1‐AP; Proteintech, Wuhan, China); Pan Phospho‐serine/threonine/tyrosine Antibody (1:500, #M210030; Abmart Co., Ltd., Shanghai). For Co‐IP, the protein was extracted from the treated neonatal mice ventricular myocytes (1 × 10^7^) or MCM cells using 0.5 mL IP lysis buffer (#87787; Thermo Fisher Scientific, Waltham, USA), supplemented with protease/phosphatase inhibitor cocktail (#78440; Thermo Fisher Scientific, Waltham, USA). Incubated on ice for 10 mins, along with vortex to disrupt properly. Then, the corresponding primary of 5 µg was added with treated 30 µL BeyoMag Protein A+G (#P2108, Beyotime Biotechnology, Shanghai, China) on a vibrator at 4 °C for 1 h. After centrifugation, the beads were collected and washed 3 times with 1 × TBS, followed by addition to the prepared protein lysate of 400 µg at 4 °C overnight on a rotary mixer. The magnetic bead mixture obtained in the previous step was washed with 1 × TBS for 3 times, followed by resuspending with IP lysis buffer containing 5X loading buffer, boiled for 5 mins at 95 °C.

### RNA Pull‐Down Assay and Liquid Chromatography‐Mass Spectrometry (LC‐MS)

Biotin‐labeled RNA Foxo6os was transcribed in vitro using the Ribo RNAmax‐T7 RNA Transcription Kit (RiboBio Co., Ltd., Guangzhou, China) and Pierce RNA 3’ End Desthiobiotinylation Kit (#20163, Thermo Fisher Scientific, Waltham, USA), according to the manufacturer's instructions. Next, the RNA–protein complex was obtained through the Pierce Magnetic RNA–protein Pull‐Down Kit (#20164, Thermo Fisher Scientific, Waltham, USA), and expanded for western blotting analysis, following the descriptions in previous studies.^[^ ^55^ ^]^ For the LC‐MS analysis, the enriched protein was resolved, washed, and enzymatically digested with sequencing‐grade modified trypsin (Promega, 50:1 enzyme‐to‐substrate ratio) at 37 °C overnight. The LC‐MS analysis was conducted using a Q Exactive mass spectrometer coupled with an Easy‐nLC 1000 system (Thermo Fisher Scientific, Waltham, MA, USA). The resulting raw data were processed using the MaxQuant 1.6.14 search engine (Bioinformatics Solutions) against the UniProt Human Proteome database (EMBL‐EBI). Peptide filtering was performed with a threshold of −10 log *P* ≥ 20, while protein identification was based on the presence of at least one unique peptide and a −10 log *P* ≥ 15.

### RNA‐Protein Interaction Modeling Workflow

First, the RNA sequence and protein sequence were uploaded to the AlphaFold 3 platform (https://golgi.sandbox.google.com/), which was utilized to generate the corresponding protein and RNA structural models. Subsequently, the obtained protein and RNA structure files were imported into the Haddock software (https://rascar.science.uu.nl/haddock2.4/) for molecular docking simulations between the protein and RNA molecules. After completion of the docking process, the docking scores generated by Haddock were used to select the model with the lowest score, representing the most likely binding conformation. Finally, the selected docking results were subjected to visualization analysis using PyMOL‐2.1, to elucidate the interaction interface and 3D structural characteristics between the protein and RNA.

### RNA Immunoprecipitation (RIP) Assay

The RIP assay was conducted employed the Magna RIP Kit (No.17‐701, Millipore, MA, USA) following the manufacturer's instructions. As described in previous studies,^[^ ^55^ ^]^ the MCM cells (2 × 10^7^) were harvested and lysed with RIP lysis buffer. Interacting RNAs were subsequently precipitated with anti‐MYBPC3. Notably, the anti‐IgG antibody (#A7028, Beyotime Biotechnology, Shanghai, China) was served as the negative control. The co‐precipitated RNAs were then isolated using Trizol and their enrichment was assessed by RT‐qPCR.

### RNA‐Fluorescence In Situ Hybridization (FISH) and IF)

To explore the subcellular distribution of Foxo6os, as well as the co‐localization between Foxo6os and MYBPC3 in the heart tissue and cardiomyocytes, the FISH was performed using Cy3‐labeled Foxo6os‐specific probe from Servicebio (Wuhan, China). As indicated, the pmCMs were washed with PBS for three times, along with fixed with 4% paraformaldehyde. After permeabilization with 0.5% Triton‐100 for 10 mins, the cells were pre‐hybridized at 55 °C for 30 min, followed by incubation at 55 °C for 1 h with the Foxo6os‐FISH probe in the hybridization buffer. Following hybridization, the sections were added with the signal probe at 42 °C for 3 h and washed in turn with 2 × SSC at 37 °C for 10 min, 1 × SSC at 37 °C for 5 min twice at dark. IF was proceeded to wash with PBST h for 3 times. Then, the sections were blocked with 3% BSA for 2 h at room temperature (RT), subsequently incubating with primary antibody (anti‐MYBPC3 1:200, anti‐α‐actinin 1:500, #11313‐2‐AP, Proteintech, Wuhan, China) at 4 °C overnight. After washing with PBST, the corresponding fluorescein‐labeled secondary antibody (#SA00013‐1, Proteintech, Wuhan, China) was added and incubated at room temperature for 1 h, then added DAPI (#C1005, Beyotime Biotechnology, Shanghai, China) to stained the nucleus for 5 mins, which were later subjected to confocal imaging. The sequence of the *Foxo6os* signal probe was as follows: GCCCCTTATACATGAACCTTTCGGCTGGCATTGATTCTCTACCTCTGTTGAAGATTCTCAGTCAGGAGTTCCGCAG.

### Histological Analysis

The heart tissue was fixed with 4% paraformaldehyde, followed by dehydrated, transparent, and embedded with paraffin. Finally, the heart tissue was cut into 6 µm thick samples for subsequent staining. The slices were stained with H&E (#GP0120, Servicebio, Wuhan, China), Masson stain (#GP1032, Wuhan, China), and WGA stain (#GDP1020, Servicebio, Wuhan, China), according to the manufacturer's instructions.

### Flow Cytometry of Apoptosis Analysis

After transfection, the adhered cells were digested from dishes and washed 3 times using PBST. Then the eBioscience Annexin V Apoptosis Detection Kit FITC (# 88‐8006‐72, Invitrogen, USA) was utilized following by the manufacturer's instructions. Briefly, diluting 10 × binding buffer to 1× using distilled water, subsequently washing cells once in ice‐cold PBS, then once in 1× binding buffer. After that, resuspended cells in 1× binding buffer at 1–5 × 10^6^ mL^−1^ and 5 µL of fluorochrome‐conjugated Annexin V was added to 100 µL of the cell suspension in concert with 5 µL of 7‐AAD Viability Staining Solution. Flow cytometry (BD FACSCantoII System, USA) was used to detect the proportion of apoptotic cells. Finally, the analysis was evaluated using the FlowJo 10.8.1 program.

### TUNEL Staining

Briefly, the heart tissues were separated and fixed in 10% phosphate‐buffered formalin for 24 h, subsequently embedded in paraffin, and sliced into 5 µm sections. TUNEL staining was performed using the Tunel Cell Apoptosis Detection Kit (#G1504‐50T, Servicebio, Wuhan, China), following manufacturer's instructions. Apoptotic nuclei were labeled with green fluorescein staining and total cardiomyocyte nuclei were marked with DAPI. The pictures of heart tissues were viewed by confocal microscopy (LSM700, Zeiss, Jena, Germany). The rate of apoptosis was displayed as the ratio of TUNEL‐positive nuclei to DAPI‐stained nuclei.

### Transmission Electron Microscopy

The hearts of the mice were perfused with 0.9% saline, and tissues separated from the left ventricle were cut into 1 mm^3^ for subsequent experiments. The samples were fixed by the pre‐cooled 2.5% glutaraldehyde and sent to Servicebio Biotechnology Co., Ltd (Wuhan) for further processing and slicing. Images were acquired under a transmission electron microscope (HT7800, HITACHI). The sarcomere length was quantified using Image J software.

### Ca^2+^ Assay

The Ca^2+^‐sensitive fluorescent probe Rhod‐2 (Yesen Biotechnology Co., Ltd., Shanghai, China) was used to detect mitochondria Ca^2+^ levels. Briefly, pmCMs were isolated and cultured in the confocal dishes and transfected with plasmids overexpressing Foxo6os. Then the cells were washed twice with PBS and incubated with 2.5 µm Rhod‐2 AM at 37 °C for 30 mins in the dark. Afterward, the cells were washed three times with PBS and subsequently incubated with PBS at 37 °C for 30 mins. Ultimately, Ca^2+^ signaling fluctuations were measured by recording the changes of fluorescence intensity during the beating of cardiomyocytes over 10 s intervals under a confocal microscope (Cytation C10, BioTek Instruments, Inc., Winooski, VT, USA). The mitochondrial Ca^2+^ concentration was reflected by the average fluorescence intensity. In each experiment, the fluorescence intensity changes of 10–20 cells were recorded on average.

### L‐type Ca ^2+^ Current (I_CaL_) Measurements

Under room temperature conditions of ≈24 °C, whole‐cell patch‐clamp experiments were performed on isolated cardiomyocytes using an Axopatch 200B amplifier provided by Axon Instruments/Molecular Devices (Sunnyvale, CA, USA). Current data recorded with 2–3  mΩ patch pipettes were normalized based on cell membrane capacitance and converted to current density units (pA pF⁻¹). For the measurement of *I*_CaL_, the pipette solution contained the following components (in mM): 120 CsCl, 6.8 MgCl_2_, 5 Na_2_ATP, 5 sodium creatine phosphate, 0.4 Na_2_GTP, 11 EGTA, 4.7 CaCl_2_ (yielding a free [Ca^2^⁺] of 120 nm), and 20 HEPES; the pH was adjusted to 7.2 with CsOH. The bath solution consisted of (in mM): 135 TEA‐Cl, 2 MgCl_2_, 10 glucose, 10 HEPES, and 1.8 CaCl_2_, with the pH adjusted to 7.4 with TEAOH. The current–voltage relationship was established by applying test pulses ranging from −80 to +60 mV from a holding potential of −80 mV. Electrophysiological data acquisition and analysis were completed using pCLAMP software (version 6, Axon Instruments/Molecular Devices).

### Cultivation of Human Embryonic Stem Cell‐Derived Cardiomyocytes (hESCs‐CMs)

The induction process of hESCs‐CM was conducted as previously described.^[^ ^56^ ^] ^Briefly, hESCs were seeded as single cells (1 × 10^5^ cm⁻^2^) on Matrigel‐coated plates in a conditioned medium with Chiron 99021 (1 µM, Cayman Chemical) and ROCK inhibitor (Y‐27632, Selleck). On day 0, the medium was replaced with RPMI+B27 containing Activin‐A (100 ng mL⁻¹) for 18 h. On day 1, cells were fed with RPMI+B27 with BMP4 (5 ng mL⁻¹) and Chiron 99021 (1 µM) for 48 h. On day 3, the medium was replaced with RPMI+B27 containing Xav 939 (1 µM). On day 5, cells were fed with RPMI+B27, and from day 7, RPMI+B27 with insulin, refreshing every other day until protocol completion.

### Human Plasma Samples

Plasma samples were collected from the peripheral venous blood of HF patients with HCM or DCM, following standardized collection and processing protocols. This study was approved by the Ethics Committee of Tongji University School of Medicine (No. 2024tjdxsy019) and conducted in accordance with the Declaration of Helsinki. Written informed consent was obtained from all participants. The design of this study is described in detail elsewhere.^[^ ^57^ ^]^ In brief, plasma samples were obtained from serial blood collections of 9 HF patients with reduced ejection fraction (HFrEF, LVEF < 40%) and 3 healthy individuals. Blood samples were collected in EDTA‐coated tubes, centrifuged at 1000 g for 10 mins and stored at −80 °C until further analysis.

### Extraction and Reverse Transcription of Plasma Exosomal RNA

Exosomes were extracted from plasma using the total exosome isolation kit (from plasma) (#4484450, Thermo Fisher Scientific, Waltham, USA) according to the manufacturer's instructions. Briefly, exosomes were enriched by brief low‐speed centrifugation. After treatment with proteinase K for 10mins, the reagent was added to the plasma, and the solution was incubated at 2–8 °C for 30 mins. The exosome pellet was then recovered by standard centrifugation (10 000 × g for 5mins) at room temperature, subsequently resuspended in PBS for further RNA extraction. Total RNA from exosomes was extracted using Thermo Fisher's TRIzol LS reagent (#10296010CN, Thermo Fisher Scientific, Waltham, USA). Briefly, 0.75 mL of TRIzol LS reagent was added to the aqueous phase, followed by the addition of 0.5 mL isopropanol. The mixture was incubated at room temperature (RT) for 10 mins and then centrifuged at 12 000 × g for 10 mins at 4 °C. The total RNA formed a white gel‐like precipitate at the bottom of the tube. The supernatant was discarded, and the pellet was resuspended in 1 mL of 75% ethanol. After a brief vortex, the solution was centrifuged at 7500 × g for 5 mins at 4 °C. The supernatant was discarded, and the RNA pellet was vacuum‐dried or air‐dried at RT for 5–10 mins for further analysis. Due to the low RNA content and enrichment of specific RNA types in exosomes, *cel‐miR‐39‐3p* (1 pmol, Tianjin Sheweisi Biotech Co., Ltd) was synthesized and added in each sample as the reference gene to enhance the accuracy and reliability of subsequent RT‐qPCR analyses. The sequence of *cel‐miR‐39‐3p* is shown as follows: UCACCGGGUGUAAAUCAGCUUG. The primers of *cel‐miR‐39‐3p* used are listed in Table S1 (Supporting Information).

### RNA‐seq Data Processing

RNA‐seq raw sequencing data (GSE112055 and GSE66630) were downloaded from the Sequence Read Archive (SRA) database using prefetch (version 2.11.0) and fastq‐dump (version 2.11.0).^[^ ^58^, ^59^ ^]^ The fastq files underwent quality control (QC) using rabbit_qc (version 0.0.1) with the parameters “‐c –detect_adapter_for_pe”.^[^ ^60^ ^]^ The human and mouse reference transcriptomes were from Ensembl release 107.^[^ ^61^ ^]^ Transcript quantification was performed using salmon (version 1.9.0) with the parameters “‐l A –seqBias –gcBias –d –posBias –hardFilter –discardOrphansQuasi –numGibbsSamples 50” on the quality‐controlled fastq files.^[^ ^62^ ^]^ Differential gene expression analysis was conducted using fishpond (version 2.8.0) in R (version 4.4.3) with default settings.^[^ ^63^ ^]^

### Statistical Analysis

The data presented in this study are representative of a minimum of three independent experiments and are expressed as mean ± standard deviation (SD). The specific group size (n) for each experiment was indicated, with ‘*n*’ representing biological replicates rather than technical replicates. To assess significant differences between groups, a two‐tailed Student's *t*‐test was employed for comparisons between two groups. A statistically significant difference was achieved at *p* < 0.05. All data analyses were conducted using GraphPad Prism version 8.0 software (San Diego, CA, USA).

## Conflict of Interest

The authors declare no conflict of interest.

## Author Contributions

J.S. and Q.L. contributed equally to this work. J.S. designed and conducted the experiments. Q.L. performed the majority of the bioinformatics analyses. Y.S., Y.M., and S.H. helped conducted experiments. F.L. and H.C. helped to the research execution. L.L. and L.P. equally conceived the study, contributed to the experimental design, and provided insights into data interpretation. All authors have reviewed the contents of the manuscript, approve of its contents, and validate the accuracy of the data.

## Supporting information



Supporting Information

Supporting Information

Supplemental Video 1

Supplemental Video 2

Supplemental Video 3

Supplemental Video 4

Supporting Table 7

Supporting Table1–6, 8

## Data Availability

The data that support the findings of this study are available from the corresponding author upon reasonable request.
