# Supplementary material for: LncRNA Foxo6os as a Novel “ Scaffold” Mediates MYBPC3 in Combating Pathological Cardiac Hypertrophy and Heart Failure
Source: Adv Sci (Weinh). 2025 Jun 23;12(34):e07365. doi: 10.1002/advs.202507365 (PMC12442697; doi:10.1002/advs.202507365)
Supplement: Supplementary file 1 — Supporting Information [file ADVS-12-e07365-s002.docx]

***Supplemental* Figure Legends**


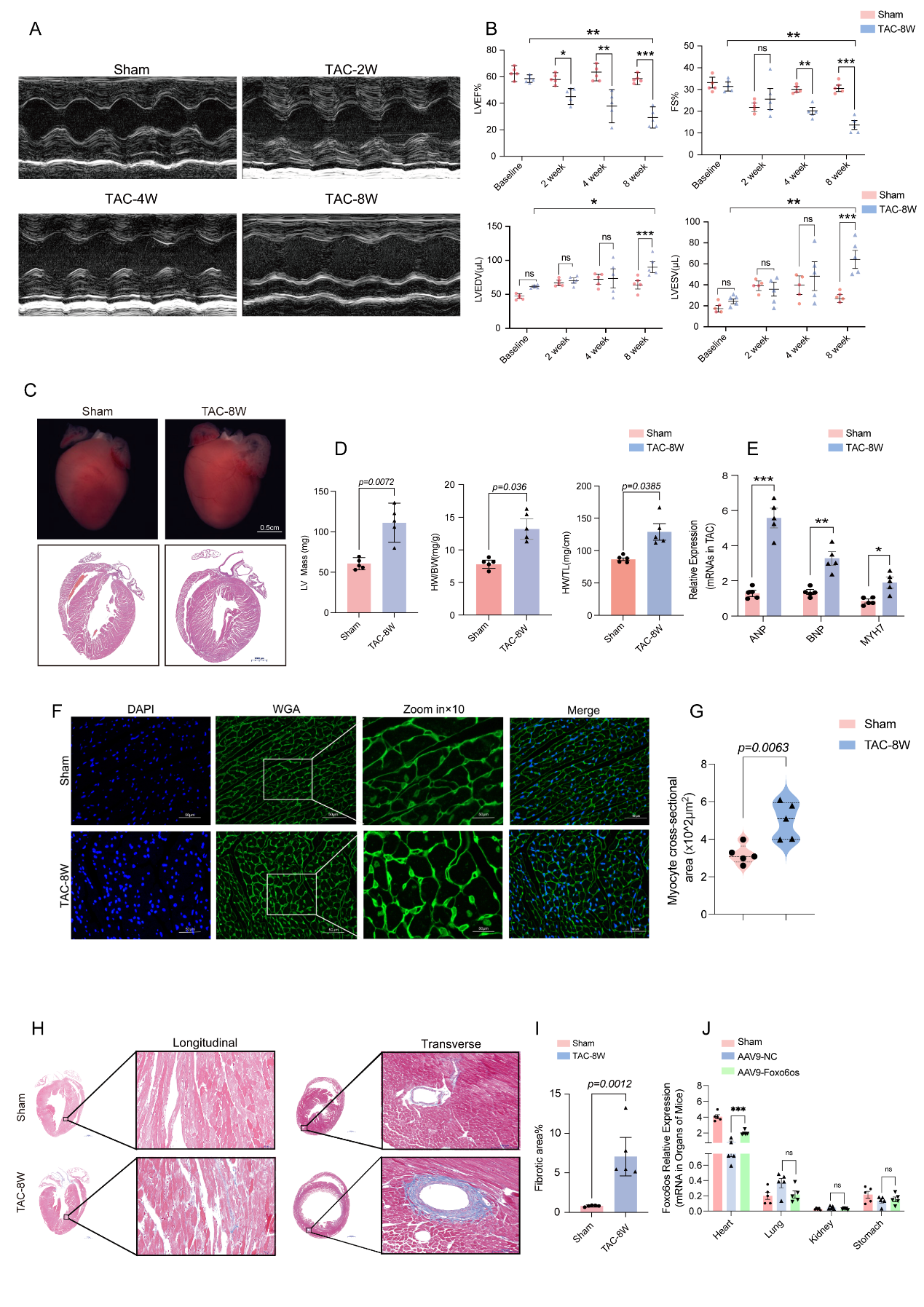
***Supplemental Figure 1***

**A)** Representative echocardiographic images of sham and TAC surgery at 2W, 4W, and 8W (n=5/group). **B)** The echo parameters of sham and TAC-surgery 8W mice were calculated at baseline, TAC-surgery 2W, 4W, 6W and 8W(n=5/group). **C)** Representative gross images of the heart (top, Scale bar=0.5cm) and hematoxylin and eosin (H&E)–stained images of cardiac morphology (bottom, Scale bar=2000μm) respectively in sham and 8W TAC-surgery group (n=5/group). **D)** Left ventricular mass (LV Mass) and heart weight normalized to body weight/tibia length (HW/BW, HW/TL) were compared between 8W TAC-surgery and sham groups (n=5/group). **E)** RT-qPCR analysis showing ANP, BNP and MYH7 expression at 8W post-TAC surgery and sham group (n=5/group). **F)** and **G)** Representative WGA staining images, partially magnified images and quantitation of cardiomyocyte sizes in 8W TAC-surgery and sham groups (scale bar = 200μm, n=5/group). **H)** Representative images, as well as partially magnified images of Masson staining for longitudinal and transverse sections of sham and TAC-surgery 8W mice. **I)** Analysis of cardiac fibrosis in **H** (scale bar = 1000μm, n=5/group). **J)** RT-qPCR analysis showing the Foxo6os expression in the organs of sham, AAV9-NC and AAV9-Foxo6os groups (n=5/group). All experiments were performed with more than three independent replicates. *p*<0.05 indicates statistical significance. ns=not significant, **p*<0.05, ***p*<0.01, ****p*<0.001. These data are presented as means±SD and analyzed using unpaired Student’s *t* test.


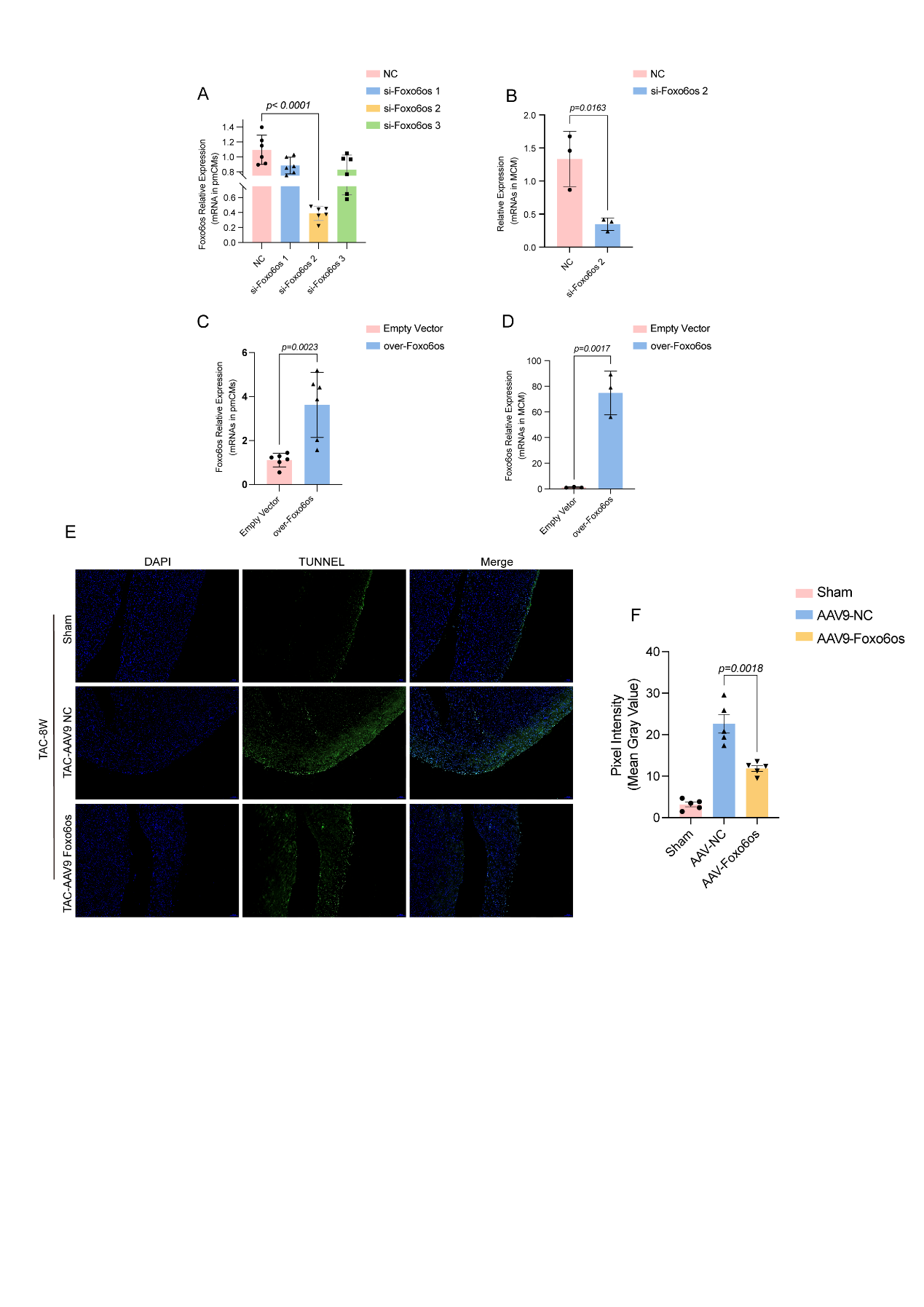
***Supplemental Figure 2***

**A)** Knockdown efficiency of three Foxo6os siRNAs was evaluated in pmCMs (n=6/group). **B)** The knockdown efficiency of si-Foxo6os2 was validated in MCM cardiomyocytes (n=3/group). **C)** and **D)** The overexpression efficiency of Foxo6os was detected respectively in pmCMs(n=6) and MCM (n=3) . **E)** TUNEL immunofluorescence staining was performed following TAC surgery 8W with AAV9-NC/Foxo6os injection (n=5/group, Scale bar=100μm). **F)** Quantification of **E** (n=5/group). All experiments were performed with more than three independent replicates. *p*<0.05 indicates statistical significance. These data are presented as means±SD and analyzed using unpaired Student’s *t* test.


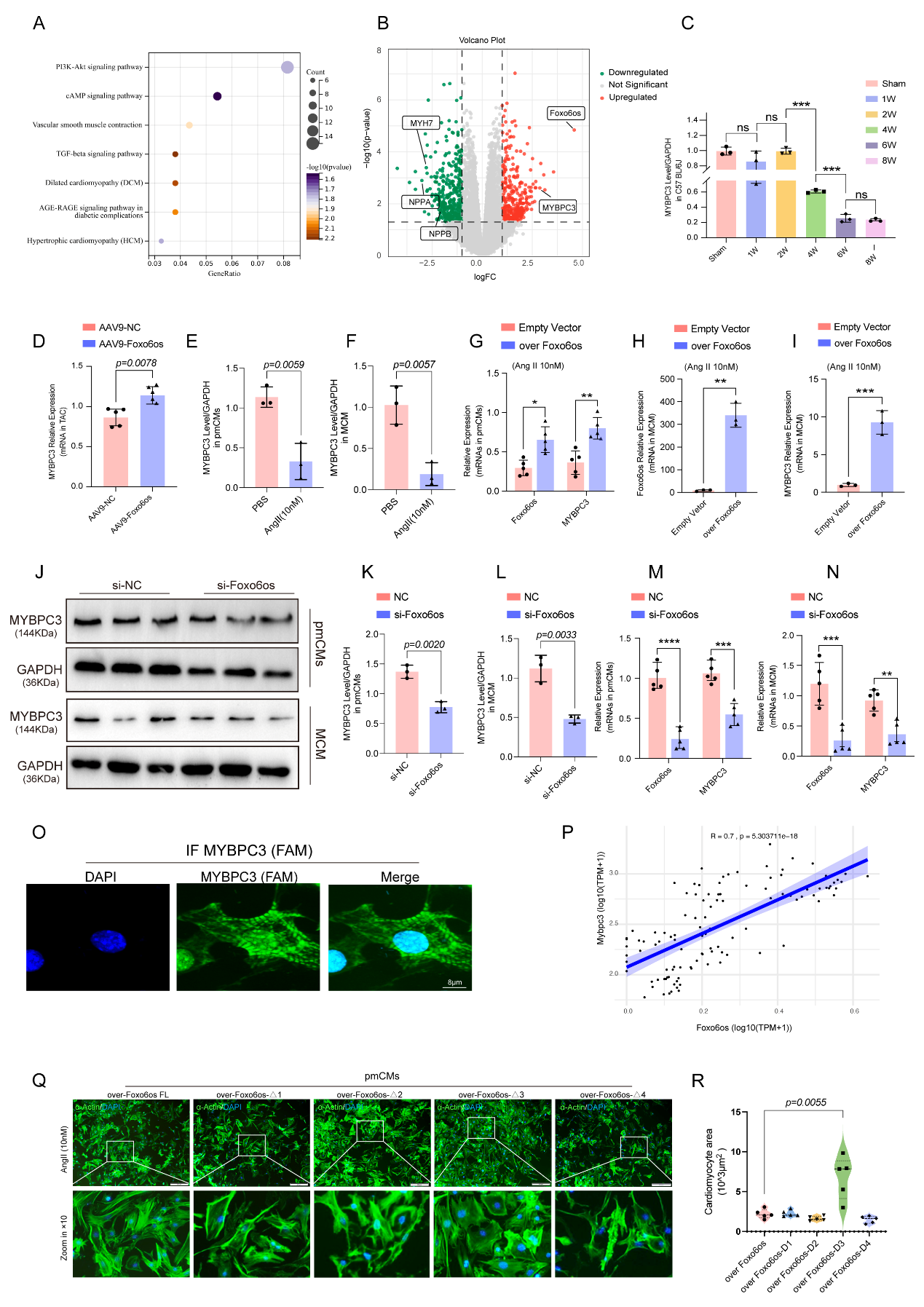
***Supplemental Figure 3***

**A)** Gene Ontology (GO) analysis of the abnormally expressed genes in AAV9-NC vs AAV9 Foxo6os mice at 8W post-TAC surgery (n=5/group). **B)** The volcano plot of cardiac differentially expressed genes analysis in AAV9-NC/Foxo6os mice at TAC-surgery 8W (n=5/group). **C)** Quantification of Figure 5A (n=3/group). **D)** Relative expression of MYBPC3 mRNA level in AAV9-NC/Foxo6os mice at TAC-surgery 8W (n=5/group). **E)** and **F)** Quantification of Figure 5B (n=3/group). **G), H)** and **I)** Relative expression of Foxo6os and MYBPC3 respectively in pmCMs (n=5)and MCM (n=3) when overexpressing Foxo6os, following treated with AngII(10nM) for 48h. **J)** Western blotting analysis of MYBPC3 expression when transfecting with Foxo6os/NC siRNAs respectively in pmCMs and MCMs (n=3/group). **K)** and **L)** Quantification of Figure S3J (n=3/group). **M)** and **N)** Relative expression of Foxo6os and MYBPC3 respectively in pmCMs and MCM when transfecting with Foxo6os/NC siRNAs (n=5/group). **O)** Immunofluorescent staining of MYBPC3 (FAM) in pmCMs (Scale bar=8μm). **P)** Pearson correlation coefficient between Foxo6os and MYBPC3 using log-transformed TPM values from 112 samples of sham and TAC mouse hearts, which were obtained from GSE112055, GSE133054, GSE134085, GSE66630, and GSE95140. **Q)** and **R)** Representative α-actin/DAPI staining, partial magnified views and the matching quantification of cardiomyocytes areas in AngII(10nM)-treated pmCMs transfecting with full-length Foxo6os, and the deletion mutants Foxo6os-△1 (592-2509 bp), Foxo6os-△2 (1-1600 bp), Foxo6os-△3 (1-592 bp), and Foxo6os-△4 (1-592 bp + 1600-2509 bp) (n=5/group). All experiments were performed with more than three independent replicates. *p*<0.05 indicates statistical significance. ns=not significant, **p*<0.05, ***p*<0.01, ****p*<0.001, *****p*<0.001. These data are presented as means±SD and analyzed using unpaired Student’s *t* test.


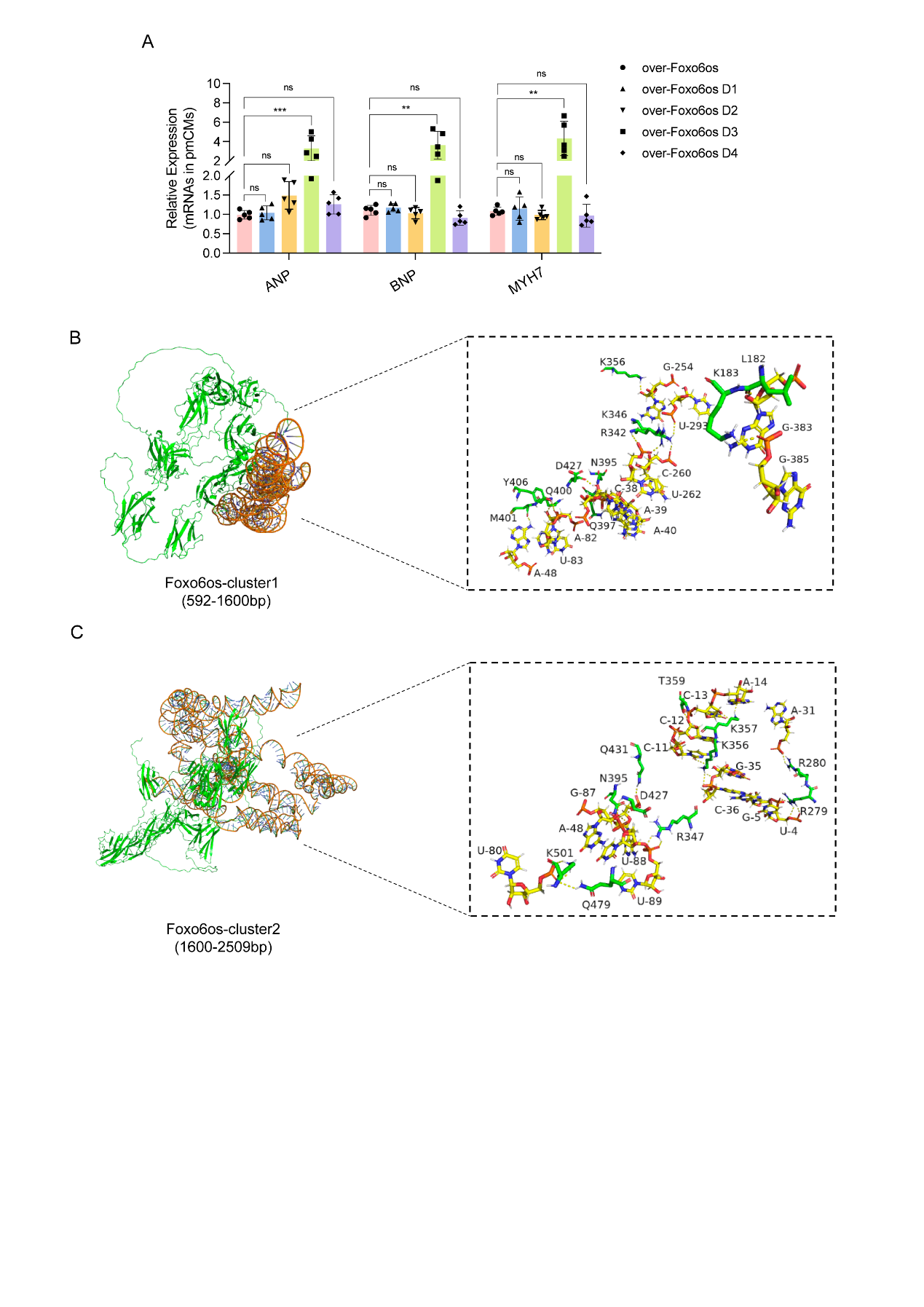
***Supplemental Figure 4***

**A)** RT-qPCR analysis of the mRNA expression levels of ANP, BNP, and MYH7 in AngII-treated pmCMs transfecting with full-length Foxo6os, and the deletion mutants Foxo6os-△1 (592-2509 bp), Foxo6os-△2 (1-1600 bp), Foxo6os-△3 (1-592 bp), and Foxo6os-△4 (1-592 bp + 1600-2509 bp) (n=5/group). **B)** Three-dimensional molecular docking models illustrating the spatial conformation of the interaction between Foxo6os and MYBPC3. All experiments were performed with more than three independent replicates. ns=not significant, ***p*<0.01, ****p*<0.001. These data are presented as means±SD and analyzed using unpaired Student’s *t* test.


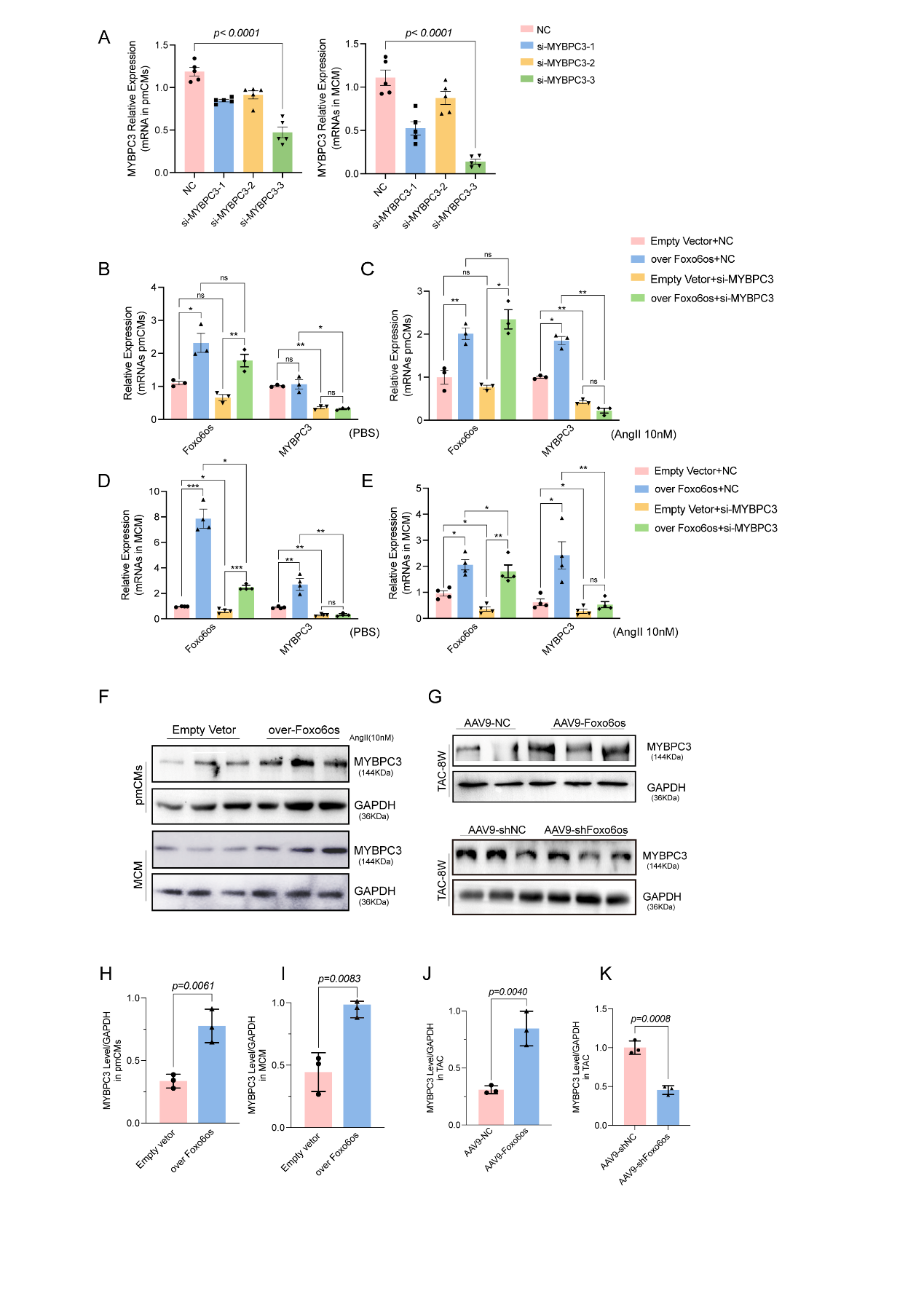
***Supplemental Figure 5***

**A)** The knockdown efficiencies of three MYBPC3 siRNAs were assessed in pmCMs (n=5/group). **B)** and **C)** RT-qPCR analysis of the mRNA expression levels of Foxo6os and MYBPC3 under PBS or AngII (10nM) treatment for 48h in pmCMs (n=3/group). **D)** and **E)** RT-qPCR analysis of the mRNA expression levels of Foxo6os and MYBPC3 under PBS or AngII (10nM) treatment for 48h in MCM (n=4/group). **F)** Western blot analysis was performed to assess the changes in MYBPC3 protein levels after AngII(10nM) treatment for 48h, following by Foxo6os overexpression respectively in pmCMs and MCMs (n=3/group). **G)** Western blot analysis was performed to detect the MYBPC3 protein levels in AAV9-NC/Foxo6os groups and AAV9-shNC/Foxo6os group at 8W post-TAC surgery. **H)** and **I)** Quantification of Figure S5F (n=3/group). **J)** and **K)** Quantification of Figure S5G (n=3/group). All experiments were performed with more than three independent replicates. **p*<0.05, ***p*<0.01, ****p*<0.001. These data are presented as means±SD and analyzed using unpaired Student’s *t* test.


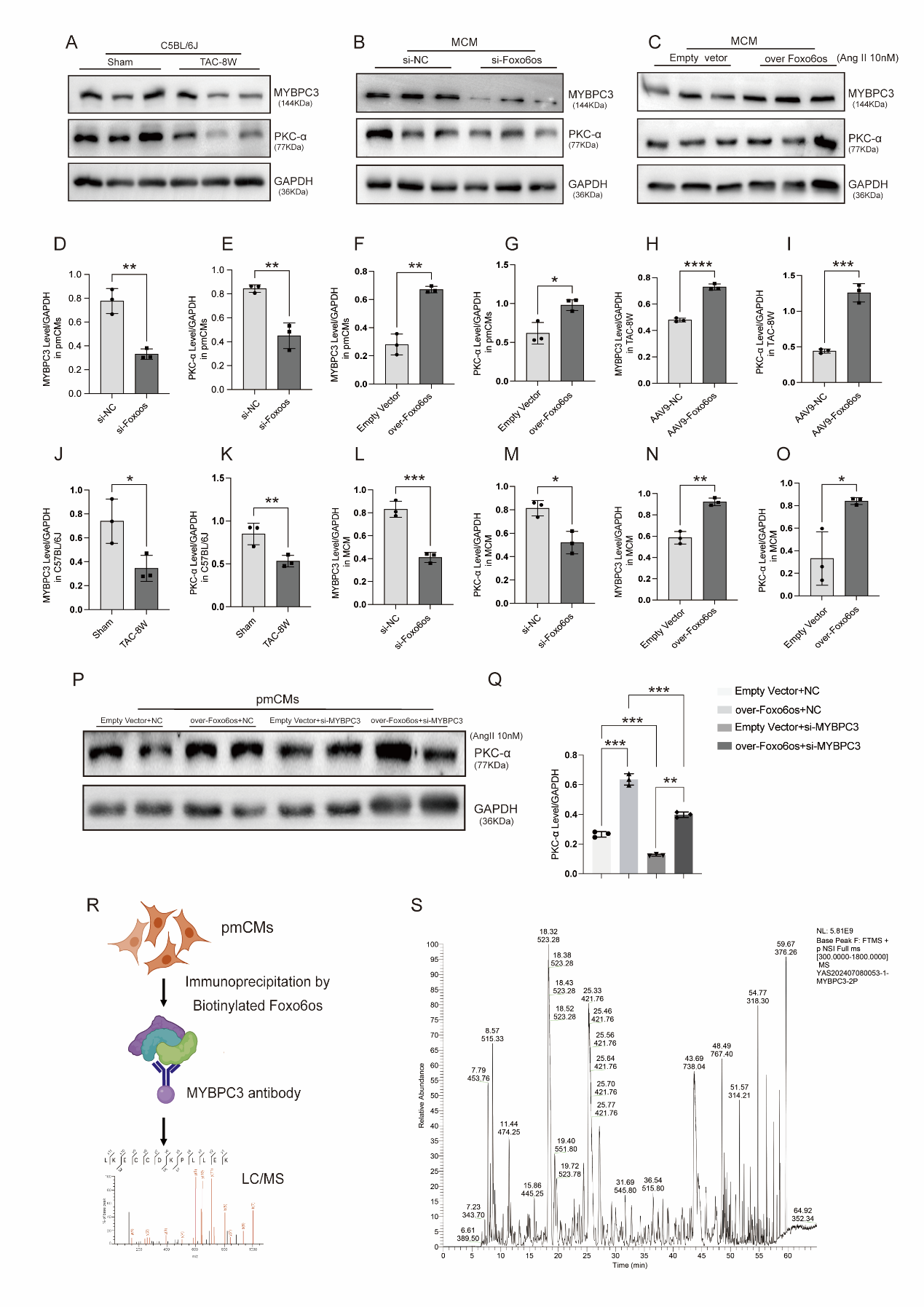
***Supplemental Figure 6***

**A)** Western blot analysis of PKC-α and MYBPC3 protein levels in sham and TAC-surgery 8W mice (n=3/group). **B)** Western blot analysis of PKC-α and MYBPC3 protein levels in MCM treated with Foxo6os/NC siRNA (n=3/group). **C)** Western blot analysis of PKC-α and MYBPC3 protein levels in MCM treated with AngII (10nM) for 48h, following by Foxo6os overexpression (n=3/group). **D)** and **E)** Quantification of Figure 7A (n=3/group). **F)** and **G)** Quantification of Figure 7B (n=3/group). **H)** and **I)** Quantification of Figure 7C (n=3/group). **J)** and **K)** Quantification of the data in **A** (n=3/group). **L)** and **M)** Quantification of the data in **B** (n=3/group). **N)** and **O)** Quantification of the data in **C** (n=3/group). **P)** Representative images of PKC-α protein levels respectively in control group, Foxo6os overexpression plasmid transfection alone, MYBPC3 siRNA transfection alone and co-transfection of Foxo6os overexpression plasmid and MYBPC3 siRNA, following by AngII (10nM) treatment for 48h. **Q)** Quantification of the data in **P** (n=3/group). **R)** Schematic for RNA-pulldown–mass spectrometry analysis. **S)** The base peak corresponding to the titanium segment of the relevant protein in the tandem mass spectrometry (LC/MS) spectrum. All experiments were performed with more than three independent replicates. **p*<0.05, ***p*<0.01, ****p* <0.001, *****p* <0.0001. These data are presented as means±SD and analyzed using unpaired Student’s *t* test.


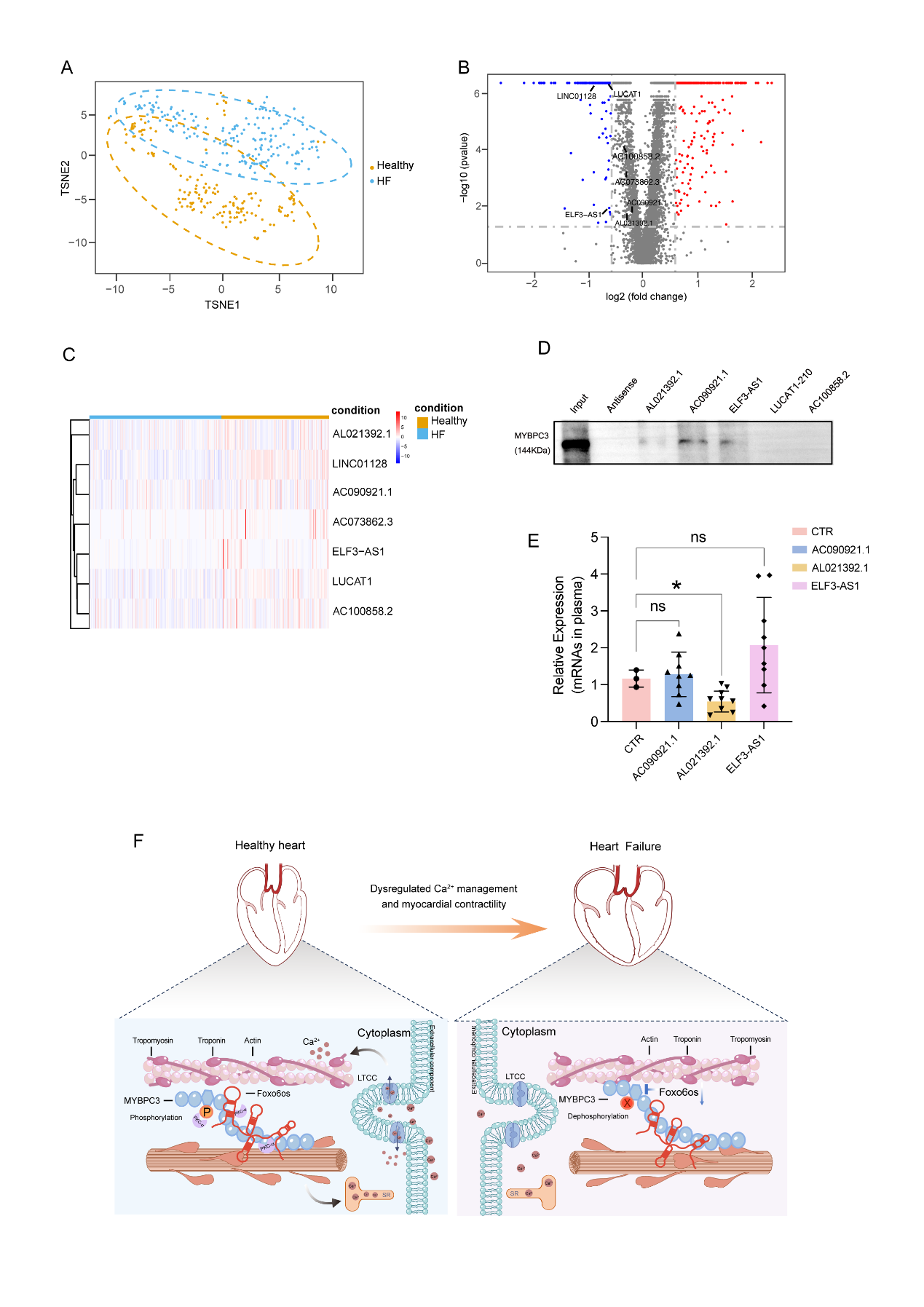
***Supplemental Figure 7***

**A)** Re-analysis of RNA-sequencing data (GSE141910) from healthy and HF human left ventricles using t-distributed stochastic neighbor embedding (t-SNE). **B)** and **C)** The volcano plot and heatmap showed differentially expressed genes (DEGs) from the samples of HF patients. **D)** RNA-pulldown analysis of MYBPC3 with function-related 5 lncRNAs in human embryonic stem cell-derived cardiomyocytes (hESCs-CMs).

**E)** RT-qPCR analysis of the mRNA expression levels of lncRNA AC090921.1, AL021392.1 and ELF3-AS1 in exosomes extracted from peripheral blood of HF patients and healthy controls (n=9 in HF group, n=3 in healthy group). **F)** Schematic illustration of the underlying mechanism in this study: Foxo6os serves as a “scaffold” within the complex, directly binding to MYBPC3 and recruiting PKC-α, thereby mediating the phosphorylation of MYBPC3 at specific sites. This post-translational modification is crucial for maintaining cardiac contraction through gating L-type calcium channels, particularly under pathological conditions of myocardial hypertrophy-induced HF. All experiments were performed with more than three independent replicates. ns=not significant, **p*<0.05. These data are presented as means±SD and analyzed using unpaired Student’s *t* test.

***Supplemental Table S1-S6, S8***

***Supplemental Video 1-4***

***Supplemental Material 1***

***Supplemental Table S7 Z-score***

| Primer Name | Sequence (5' to 3') |
| --- | --- |
| Foxo6os Forward primer | TCCCTTTCCATCCTGCCTTCT |
| Foxo6os Reverse primer | TTCATTTGCCCCGCACTTCTT |
| GAPDH Forward primer | TTTGCAGTGGCAAAGTGGAGATT |
| GAPDH Reverse primer | CCCATTTGATGTTAGTGGGGTCTCG |
| MYBPC3 Forward primer | GCTGGCTCTGTCATAGCTGTC |
| MYBPC3 Reverse primer | TGGCCTCTTTCTGATGCGAC |
| ANP Forward primer | GCTTCCAGGCCATATTGGAG |
| ANP Reverse primer | GGGGGCATGACCTCATCTT |
| BNP Forward primer | AGTCCTTCGGTCTCAAGGCA |
| BNP Reverse primer | CCGATCCGGTCTATCTTGTGC |
| MYH7 Forward primer | CCTGCGGAAGTCTGAGAAGG |
| MYH7 Reverse primer | CTCGGGACACGATCTTGGC |
| cel-miR-39-3p Forward primer | GGCGACCGGGTGTAAATCA |
| cel-miR-39-3p Reverse primer | AGTGCAGGGTCCGAGGTATT |

***Supplemental Table S1 the Sequences of Primers***

***Supplemental Table S2 the Sequences of siRNAs***

***Supplemental Table S3 the Subcellular Localization of Foxo6os Predicated by LncLocator and DeepLncLoc***

| siRNAs | Sequence (5' to 3') |
| --- | --- |
| si-Foxo6os1 | GCAGATGGAACTAAGTATA |
| si-Foxo6os2 | CAAGTAGCTACGGAGAGTT |
| si-Foxo6os3 | CGCAATGACCATACACTCA |
| si-MYBPC3-1 | GCAGCAAGTACATCTTCGA |
| si-MYBPC3-2 | GCATAAAGGTGTCCCATAT |
| si-MYBPC3-3 | CTGCTGAAGAAGAGAGACA |

| **LncRNA** | **Algorithm** | **Cytoplasm** | **Nucleus** | **Ribosome** | **Cytosol** | **Exosome** |
| --- | --- | --- | --- | --- | --- | --- |
| Foxo6os | LncLocator | 0.792 | 0.164 | 0.008 | 0.029 | 0.007 |
| Foxo6os | DeepLncKoc | 0.429 | 0.36 | 0.097 | 0.089 | 0.025 |

***Supplemental Table S4 the Correlation Between Foxo6os and Mybpc3 Gene Expression***

| LncRNA | Gene | Dataset | Pearson Correlation Coefficient |
| --- | --- | --- | --- |
| Foxo6os | Mybpc3 | GeneFriends2021 | 0.539 |
| Foxo6os | Mybpc3 | GSE66630&GSE112055 | 0.697 |

***Supplemental Table S5 the Detailed Information Regarding the Docking Sites and Their Interaction Types (Foxo6os-cluster 1)***

| **MYBPC3** | **Foxo6os-cluster 1(1-1000)** | | **Interaction Types** |
| --- | --- | --- | --- |
| M401, Y406, Q400 | | A-48, U-83, A-82 | Hydrogen Bond |
| D427, N395, Q397 | | A-40, A-39, C-38 | Hydrogen Bond |
| K356, K346, R342 | | G-254, U-293, C-260, U-262 | Hydrogen Bond |
| K183, L182 | | G-383, G-385 | Hydrogen Bond |

***Supplemental Table S6 the Detailed Information Regarding the Docking Sites and Their Interaction Types (Foxo6os-cluster 2)***

| **MYBPC3** | **Foxo6os-cluster2(1600-2509)** | **Interaction Types** |
| --- | --- | --- |
| K501, Q479 | U-80 | Hydrogen Bond |
| Q431, N395, D427, R347 | A-48, G-87, U-88, U-89 | Hydrogen Bond |
| T359, K357, K356, R280, R279 | A-14, A-31, C-13, C-12, C-11, C-36, G-35, G-5, U-4 | Hydrogen Bond |

***Supplemental Table S8 the Correlation Z-score of Foxo6os with Function-related LncRNAs***

|  | GENEID | Z-score |
| --- | --- | --- |
| ELF3-AS1 | ENSG00000234678 | 2.534 |
| LUCAT1 | ENSG00000248323 | 2.247 |
| AC1000858.2 | ENSG00000255491 | 2.004 |
| AL021392.1 | ENSG00000234869 | 2.895 |
| AC090921.1 | ENSG00000214803 | 2.237 |
