# Supplementary material for: LncRNA Foxo6os as a Novel “ Scaffold” Mediates MYBPC3 in Combating Pathological Cardiac Hypertrophy and Heart Failure
Source: Adv Sci (Weinh). 2025 Jun 23;12(34):e07365. doi: 10.1002/advs.202507365 (PMC12442697; doi:10.1002/advs.202507365)
Supplement: Supplementary file 2 — Supporting Information [file ADVS-12-e07365-s007.pdf]

ADVANCED SCIENCE
Open Access

Supporting Information

for *Adv. Sci.*, DOI 10.1002/advs.202507365

LncRNA Foxo6os as a Novel " Scaffold" Mediates MYBPC3 in Combating Pathological Cardiac Hypertrophy and Heart Failure

Jie Sheng, Qin Lin, Yizhuo Sun, Yilei Meng, Sangyu Hu, Huaming Cao, Fang Lin, Yuping Zhu, Luying Peng and Li Li**

Mouse Cardiac Myocytes (MCM)

Catalog #M6200-57

Cell Specification

Cardiac myocytes are the most physically energetic cells in the body. They are highly specialized high-oxygen-content cells that house a large number of mitochondria [1]. They occupy as much as 75% of the cardiac mass, but constitute only about one third of the total cell number in the heart. Differentiated cardiac myocytes have little capacity to proliferate; however, hypertrophic growth has been shown to respond to alpha1-adrenergic stimuli via the Ras/MEK pathway [2]. All cardiac myocytes are capable of spontaneous rhythmic depolarization and repolarization of their membranes. Contraction of cardiac myocytes is myogenic, which is independent of nervous stimulation. There is a complex network of signals in cardiac myocytes regulating the rhythmic pumping of the heart [3]. Cardiac myocyte hypertrophy and apoptosis have been implicated in the loss of contractile function during heart failure. A better understanding of the cardiac signaling network will help reveal the cellular mechanisms leading to cardiac myocyte death.

MCM from ScienCell Research Laboratories are isolated from postnatal day 2 C57BL/6 mouse heart. MCM are cryopreserved at P0 and delivered frozen. Each vial contains >1 x 10^6 cells in 1 ml volume. MCM are characterized by immunofluorescence with antibodies specific to smooth muscle actin, sacromeric alpha-actinin, and tropomyosin. MCM are negative for mycoplasma, bacteria, yeast, and fungi. MCM are guaranteed to further culture under the conditions provided by ScienCell Research Laboratories; *however, MCM are not recommended for expanding or long-term cultures since the cells do not proliferate in culture*.

Recommended Medium

It is recommended to use Cardiac Myocyte Medium-serum free (CMM-sf, Cat. #6101) for culturing MCM *in vitro*.

Product Use

MCM are for research use only. They are not approved for human or animal use, or for application in *in vitro* diagnostic procedures.

Storage

Upon receiving, directly and immediately transfer the cells from dry ice to liquid nitrogen and keep the cells in liquid nitrogen until they are needed for experiments.

Shipping

Dry ice.

References

[1] Bodyak N, Kang PM, Hiromura M, Sulijoadikusumo I, Horikoshi N, Khrapko K, Usheva A. (2002) “Gene expression profiling of the aging mouse cardiac myocytes.” *Nucleic Acids Research*. 30: 3788-94.

[2] Tamamori-Adachi M, Ito H, Nobori K, Hayashida K, Kawauchi J, Adachi S, Ikeda MA, Kitajima S. (2002) “Expression of cyclin D1 and CDK4 causes hypertrophic growth of cardiomyocytes in culture: a possible implication for cardiac hypertrophy.” *Biochem Biophys Res Commun*. 296: 274-80.

[3] Sambrano GR, Fraser I, Han H, Ni Y, O'Connell T, Yan Z, Stull JT. (2002) “Navigating the signaling network in mouse cardiac myocytes.” *Nature*. 420: 712-4.

Instructions for culturing cells

Caution: Cryopreserved cells are very delicate. Thaw the vial in a 37°C water bath and return the cells to culture as quickly as possible with minimal handling!

Initiating the culture:

1. Prepare a poly-L-lysine-coated culture vessel (2 μg/cm^2, T-75 flask is recommended). Add 10 ml of sterile water to a T-75 flask and then add 15 μl of poly-L-lysine stock solution (10 mg/ml, Cat. #0413). Leave the vessel in a 37°C incubator overnight (or for a minimum of one hour).
2. Prepare complete medium. Decontaminate the external surfaces of medium bottle and medium supplement tubes with 70% ethanol and transfer them to a sterile field. Aseptically transfer supplement to the basal medium with a pipette. Rinse the supplement tube with medium to recover the entire volume.
3. Rinse the poly-L-lysine-coated vessel twice with sterile water and then add 15 ml of complete medium. Leave the vessel in the sterile field and proceed to thaw the cryopreserved cells.
4. Place the frozen vial in a 37°C water bath. Hold and rotate the vial gently until the contents completely thaw. Promptly remove the vial from the water bath, wipe it down with 70% ethanol, and transfer it to the sterile field.
5. Carefully remove the cap without touching the interior threads. Gently resuspend and dispense the contents of the vial into the equilibrated, poly-L-lysine-coated culture vessel. A seeding density of 5,000 cells/cm^2 is recommended.

   Note: Dilution and centrifugation of cells after thawing are not recommended since these actions are more harmful to the cells than the effect of residual DMSO in the culture. It is also important that cells are plated in poly-L-lysine-coated culture vessels to promote cell attachment.
6. Replace the cap or lid of the culture vessel and gently rock the vessel to distribute the cells evenly. Loosen cap, if necessary, to allow gas exchange.
7. Return the culture vessel to the incubator.
8. For best results, do not disturb the culture for at least 16 hours after the culture has been initiated. Refresh culture medium the next day to remove residual DMSO and unattached cells, then every other day thereafter.

Maintaining the culture:

1. Refresh supplemented culture medium the next morning after establishing a culture from cryopreserved cells.
2. Change the medium every three days thereafter.

MCM are not recommended to be subcultured since this cell type will terminally differentiate in long-term cultures.

Caution: Handling animal derived products is potentially biohazardous. Always wear gloves and safety glasses when working with these materials. Never mouth pipette. We recommend following the universal procedures for handling products of animal origin as the minimum precaution against contamination [1].

[1] Grizzle WE, Polt S. (1988) "Guidelines to avoid personal contamination by infective agents in research laboratories that use human tissues." *J Tissue Cult Methods*. 11: 191-9.
