# Supplementary material for: LncRNA Foxo6os as a Novel “ Scaffold” Mediates MYBPC3 in Combating Pathological Cardiac Hypertrophy and Heart Failure
Source: Adv Sci (Weinh). 2025 Jun 23;12(34):e07365. doi: 10.1002/advs.202507365 (PMC12442697; doi:10.1002/advs.202507365)
Supplement: Supplementary file 8 — Supporting Table1–6, 8 [file ADVS-12-e07365-s001.docx]

| Primer Name | Sequence (5' to 3') |
| --- | --- |
| Foxo6os Forward primer | TCCCTTTCCATCCTGCCTTCT |
| Foxo6os Reverse primer | TTCATTTGCCCCGCACTTCTT |
| GAPDH Forward primer | TTTGCAGTGGCAAAGTGGAGATT |
| GAPDH Reverse primer | CCCATTTGATGTTAGTGGGGTCTCG |
| MYBPC3 Forward primer | GCTGGCTCTGTCATAGCTGTC |
| MYBPC3 Reverse primer | TGGCCTCTTTCTGATGCGAC |
| ANP Forward primer | GCTTCCAGGCCATATTGGAG |
| ANP Reverse primer | GGGGGCATGACCTCATCTT |
| BNP Forward primer | AGTCCTTCGGTCTCAAGGCA |
| BNP Reverse primer | CCGATCCGGTCTATCTTGTGC |
| MYH7 Forward primer | CCTGCGGAAGTCTGAGAAGG |
| MYH7 Reverse primer | CTCGGGACACGATCTTGGC |
| cel-miR-39-3p Forward primer | GGCGACCGGGTGTAAATCA |
| cel-miR-39-3p Reverse primer | AGTGCAGGGTCCGAGGTATT |

***Supplemental Table S1 the Sequences of Primers***

***Supplemental Table S2 the Sequences of siRNAs***

***Supplemental Table S3 the Subcellular Localization of Foxo6os Predicated by LncLocator and DeepLncLoc***

| siRNAs | Sequence (5' to 3') |
| --- | --- |
| si-Foxo6os1 | GCAGATGGAACTAAGTATA |
| si-Foxo6os2 | CAAGTAGCTACGGAGAGTT |
| si-Foxo6os3 | CGCAATGACCATACACTCA |
| si-MYBPC3-1 | GCAGCAAGTACATCTTCGA |
| si-MYBPC3-2 | GCATAAAGGTGTCCCATAT |
| si-MYBPC3-3 | CTGCTGAAGAAGAGAGACA |

| **LncRNA** | **Algorithm** | **Cytoplasm** | **Nucleus** | **Ribosome** | **Cytosol** | **Exosome** |
| --- | --- | --- | --- | --- | --- | --- |
| Foxo6os | LncLocator | 0.792 | 0.164 | 0.008 | 0.029 | 0.007 |
| Foxo6os | DeepLncKoc | 0.429 | 0.36 | 0.097 | 0.089 | 0.025 |

***Supplemental Table S4 the Correlation Between Foxo6os and Mybpc3 Gene Expression***

| LncRNA | Gene | Dataset | Pearson Correlation Coefficient |
| --- | --- | --- | --- |
| Foxo6os | *Mybpc3* | GeneFriends2021 | 0.539 |
| Foxo6os | *Mybpc3* | GSE66630&GSE112055 | 0.697 |

***Supplemental Table S5 the Detailed Information Regarding the Docking Sites and Their Interaction Types (Foxo6os-cluster 1)***

| **MYBPC3** | **Foxo6os-cluster 1(1-1000)** | | **Interaction Types** |
| --- | --- | --- | --- |
| M401, Y406, Q400 | | A-48, U-83, A-82 | Hydrogen Bond |
| D427, N395, Q397 | | A-40, A-39, C-38 | Hydrogen Bond |
| K356, K346, R342 | | G-254, U-293, C-260, U-262 | Hydrogen Bond |
| K183, L182 | | G-383, G-385 | Hydrogen Bond |

***Supplemental Table S6 the Detailed Information Regarding the Docking Sites and Their Interaction Types (Foxo6os-cluster 2)***

| **MYBPC3** | **Foxo6os-cluster2(1600-2509)** | **Interaction Types** |
| --- | --- | --- |
| K501, Q479 | U-80 | Hydrogen Bond |
| Q431, N395, D427, R347 | A-48, G-87, U-88, U-89 | Hydrogen Bond |
| T359, K357, K356, R280, R279 | A-14, A-31, C-13, C-12, C-11, C-36, G-35, G-5, U-4 | Hydrogen Bond |

***Supplemental Table S8 the Correlation Z-score of Foxo6os with Function-related LncRNAs***

|  | GENEID | Z-score |
| --- | --- | --- |
| ELF3-AS1 | ENSG00000234678 | 2.534 |
| LUCAT1 | ENSG00000248323 | 2.247 |
| AC1000858.2 | ENSG00000255491 | 2.004 |
| AL021392.1 | ENSG00000234869 | 2.895 |
| AC090921.1 | ENSG00000214803 | 2.237 |
